# Assigning breed origin to alleles in crossbred animals

**DOI:** 10.1186/s12711-016-0240-y

**Published:** 2016-08-22

**Authors:** Jérémie Vandenplas, Mario P. L. Calus, Claudia A. Sevillano, Jack J. Windig, John W. M. Bastiaansen

**Affiliations:** 1 Animal Breeding and Genomics Centre, Wageningen UR Livestock Research, 6700 AH Wageningen, The Netherlands; 2 Topigs Norsvin Research Center B.V., 6640 AA Beuningen, The Netherlands; 3 Animal Breeding and Genomics Centre, Wageningen University, 6700 AH Wageningen, The Netherlands

## Abstract

**Background:**

For some species, animal production systems are based on the use of crossbreeding to take advantage of the increased performance of crossbred compared to purebred animals. Effects of single nucleotide polymorphisms (SNPs) may differ between purebred and crossbred animals for several reasons: (1) differences in linkage disequilibrium between SNP alleles and a quantitative trait locus; (2) differences in genetic backgrounds (e.g., dominance and epistatic interactions); and (3) differences in environmental conditions, which result in genotype-by-environment interactions. Thus, SNP effects may be breed-specific, which has led to the development of genomic evaluations for crossbred performance that take such effects into account. However, to estimate breed-specific effects, it is necessary to know breed origin of alleles in crossbred animals. Therefore, our aim was to develop an approach for assigning breed origin to alleles of crossbred animals (termed BOA) without information on pedigree and to study its accuracy by considering various factors, including distance between breeds.

**Results:**

The BOA approach consists of: (1) phasing genotypes of purebred and crossbred animals; (2) assigning breed origin to phased haplotypes; and (3) assigning breed origin to alleles of crossbred animals based on a library of assigned haplotypes, the breed composition of crossbred animals, and their SNP genotypes. The accuracy of allele assignments was determined for simulated datasets that include crosses between closely-related, distantly-related and unrelated breeds. Across these scenarios, the percentage of alleles of a crossbred animal that were correctly assigned to their breed origin was greater than 90 %, and increased with increasing distance between breeds, while the percentage of incorrectly assigned alleles was always less than 2 %. For the remaining alleles, i.e. 0 to 10 % of all alleles of a crossbred animal, breed origin could not be assigned.

**Conclusions:**

The BOA approach accurately assigns breed origin to alleles of crossbred animals, even if their pedigree is not recorded.

**Electronic supplementary material:**

The online version of this article (doi:10.1186/s12711-016-0240-y) contains supplementary material, which is available to authorized users.

## Background

Several production systems, including those for pigs and chickens, are based on crossbreeding (e.g., [1–3]) to take advantage of the increased performance of crossbred compared to purebred animals. One limitation of these breeding programs is that selection is performed on purebred animals, although the aim is to improve crossbred performance. Besides the genetic differences between purebred and crossbred animals, purebred animals are mainly housed in nucleus farms with high-health conditions, while crossbred animals are housed under field conditions.

With the advent of genomic selection, several authors have proposed genomic evaluation methods that use phenotypic records on crossbred animals to increase response to selection for crossbred performance (e.g., [2, 4, 5]). These approaches compute estimated breeding values for crossbred performance using many single nucleotide polymorphisms (SNPs). Several factors have an impact on the effect that can be measured for a SNP. First, the effect of the same allele, but of different breed origin, in a crossbred animal may differ because of different levels of linkage disequilibrium (LD) between the SNP and a quantitative trait locus (QTL) in the purebred populations. Second, different genetic backgrounds, e.g., dominance, or epistatic interactions, can explain that the same allele has different effects in purebred and crossbred animals. Third, the environmental conditions under which purebred and crossbred animals are raised may vary, which can result in genotype-by-environment interactions. Thus, SNP effects may be breed-specific, which has led to the implementation of genomic selection of purebred animals for crossbred performance that take breed-specific effects of SNP alleles into account [3, 5]. However, these methods assume that breed origin of alleles in crossbred animals is known. Results from simulations showed that models that consider breed-specific effects can outperform the current genomic models that assume that the SNP effect is the same across breeds, at least under some conditions [2, 5]. Although breed-specific effect models appear promising based on these simulation studies, the question whether they will outperform other models remains open. To apply a model that considers breed-specific effects on real field data, accurate estimates of local ancestry for the SNP alleles of crossbred animals are needed. In this context, local ancestry refers to the breed origin of each SNP allele for each locus for each crossbred animal.

Several approaches (e.g., [6–9]) have been proposed to estimate local ancestry in admixed populations. These approaches can be an essential step in the mapping of disease genes [10], in the control of population structure for genome-wide association studies (GWAS) [11], or even in the study of population genetic processes that involve admixed populations [12–14]. Some of these approaches specifically focus on local ancestry inference in admixed populations that originate from two or more populations a few generations back. However, these approaches may be less applicable in our context for several reasons. One reason is that they do not consider that each crossbred animal originates from a well-defined crossbreeding scheme, in which the purebred populations, i.e. the ancestral populations, are at most the second ancestral generation. Also, these methods implicitly assume genetically-diverged populations [7, 8], which is generally not the case for purebred pig or chicken populations, which may include different lines of the same breed, or a cross of several breeds (i.e., a synthetic breed). Therefore, the aim of our study was to develop an approach for assigning breed origin to alleles (termed BOA) of animals that come from specific crossbreeding schemes. Furthermore, we determined the accuracy of allele assignments by using simulated datasets that involved crosses between closely-related, distantly-related or unrelated breeds. The BOA approach requires several phasing analyses of the genotypes of purebred and crossbred animals. The effects of different phasing parameters and several nuisance factors in the data, such as the presence of a haplotype in another pure breed that would preclude the assignment to the first pure breed, were also tested. In addition, the developed method was applied to real pig genotype data to investigate whether the results were consistent with those obtained from simulated data.

## Methods

### Ethics statement

The data used in this study was collected as part of routine data recording in a commercial breeding program. Samples collected for DNA extraction were only used for routine diagnostic purposes of the breeding program. Data recording and sample collection were conducted strictly in line with the Dutch law on the protection of animals (Gezondheids—en welzijnswet voor dieren).

### Simulated data

#### Populations

To test the accuracy of an approach aimed at assigning breed origin to alleles, the true origin of each allele of crossbred animals must be known. This was achieved by simulating historic and breed populations using the QMSim software [15], and then simulating a three-way crossbreeding program with five generations of random selection using a custom Fortran program. For the historic population, 1000 discrete random mating generations with a constant size of 1000 individuals were simulated, followed by 50 generations in which the effective population size was reduced to 100 individuals. The next eight generations were simulated to expand the population size to 810. For the first 1050 simulated generations, half of the simulated animals were males and the other half were females. In the next eight generations, 60 males and 750 females were simulated. Matings for all generations were based on the random union of gametes, which were randomly sampled from the pools of male and female gametes. To simulate the three breed populations (hereafter referred to as breeds A, B, and C), three random samples were drawn from the last generation of the historic population (i.e., generation 1058), each including 20 males and 250 females. Subsequently, within each breed, 5, 20, or 50 generations of random mating were simulated before starting the three-way crossbreeding scheme, which will be referred to as scenarios with closely-related breeds, distantly-related breeds, and unrelated breeds, respectively. For the simulated 5, 20, and 50 generations of pseudo-random mating, one litter with two individuals per female (i.e. one male and one female) was assumed.

In the second step, a three-way crossbreeding program with five generations of random selection was simulated. Purebred (i.e., A, B, and C) animals that were used to start the crossbreeding program were from generations 1063, 1078, and 1108 for the closely-related, distantly-related and unrelated breeds, respectively. During the crossbreeding program, and for each breed, A, B, and C, purebred animals were randomly selected and mated to simulate the next generation by maintaining a constant size of 20 males and 250 females. From each of the five generations, B and C purebred animals were randomly crossed to produce five generations of 10 BC crossbred males and 100 BC crossbred females. These BC crossbred animals were then randomly mated to males from breed A to produce five generations of A(BC) crossbred animals. For each generation, 110 A(BC) animals were simulated. Purebred animals that were used as parents of crossbred animals could also be parents of purebred animals in the next generation.

#### Genotypes

For the three scenarios, the genome consisted of two chromosomes, i.e. a 3.20 Morgan long chromosome (chr1) with 6700 SNPs and a 0.61 Morgan long chromosome (chr2) with 1353 SNPs. These two chromosomes were designed to resemble *Sus Scrofa* chromosomes (SSC) 1 and SSC18, respectively, with a SNP density that was comparable to that of a 60 k SNP chip. The SNP positions were randomized across the genome and a recurrent mutation rate of 2.5 × 10^−5^ was assumed. All SNPs that segregated in the last historical generation (i.e., generation 1058) and with a minor allele frequency (MAF) higher than or equal to 0.10 were selected and used to simulate the genotypes of the purebred and crossbred animals, as well as for all subsequent analyses. Breed origin of each allele was recorded for each crossbred animal.

To compose the datasets of genotypes, 75 % of purebred (A, B, and C) and crossbred [BC, and A(BC)] males and females that were produced during the three-way crossbreeding program were randomly selected. Random selection of purebred and crossbred animals led to datasets of genotypes that did not include all parents of the crossbred animals and for which not all purebred animals had crossbred offspring. It was assumed that pedigree information was not available for any animal.

### Real data

A total of 5692 pigs from three purebred populations (herein referred to as breeds D, E, and F) and two crossbred populations [hereafter referred to as EF (E × F or F × E) and D(EF) (D × EF)] were genotyped with the Illumina PorcineSNP60 Beadchip [16]. Breeds D, E, and F refer to a synthetic boar line, a Landrace line, and a Large White line, respectively. SNPs on SSC2 and 18 with a call rate higher than 0.95 for each purebred or crossbred population were selected. No threshold was used for MAF. Animals’ genotypes with a call rate higher than 0.98 were included for analysis. The final genotype dataset contained 2695 SNPs for SSC2 and 1129 SNPs for SSC18 that were used to genotype 956 D, 1816 E, and 1918 F purebred animals. Genotypes of 324 EF and 241 D(EF) crossbred animals were also included (Table 1).

**Table 1:** Descriptive statistics for three simulated scenarios (10 replicates; SD within brackets) and for the real data

Parameters	Scenario			
	Closely-related breeds	Distantly-related breeds	Unrelated breeds	Real dataset
Number of animals				
Purebred (A/D)	1004.0			956
Purebred (B/E)	1008.0			1816
Purebred (C/F)	993.0			1918
Crossbred (BC/EF)	414.0			324
Crossbred (A(BC)/D(EF))	428.0			241
Number of SNPs				
Chromosome 1	4800.1 (70.0)	4803.2 (68.2)	4830.7 (86.5)	–
Chromosome 2	920.2 (50.5)	949.8 (58.2)	908.8 (41.2)	–
SSC2	–	–	–	2496
SSC18	–	–	–	1129
*F*_ST_^a^	0.05 (0.00)	0.13 (0.01)	0.28 (0.02)	0.15

^a^Global Wright’s *F*_ST_ statistics

### Genetic differentiation

For the three scenarios, i.e. closely-related breeds, distantly-related breeds, and unrelated breeds, the level of genetic differentiation between the three breeds was measured using the global Wright’s *F*_ST_ statistic [17], as implemented in the software Genepop (4.2) [18, 19]. Genotypes for all selected SNPs and for all purebred animals, from all five purebred generations simulated for the three-way crossbreeding program were used to estimate *F*_ST_. The same statistics were computed for the real dataset by considering all selected SNPs on SSC2 and 18 for all available purebred animals.

### Assignment of allele origin

The BOA approach that we developed to assign breed origin to alleles of crossbred animals, consisted of three steps: (1) phasing the genotypes of both purebred and crossbred animals, (2) assigning breed origin to the phased haplotypes, and (3) assigning breed origin to alleles of crossbred animals based on the library of assigned haplotypes, the breed composition of the crossbred animals and the zygosity (i.e., homozygosity or heterozygosity) of their genotypes.

#### Phasing

AlphaPhase1.1 (version 1) software [20] was chosen for phasing available genotypes. AlphaPhase1.1 implements a long-range phasing (LRP) and haplotype library imputation algorithm (LRPHLI) and resolves phase without depending on family structure or pedigree information. The LRPHLI uses long haplotypes and the principle of surrogate parents, which are individuals that share a haplotype with the individual being phased. They are identified by having no opposing homozygote genotypes with this individual within a string of consecutive SNPs that includes a core and adjacent tails (hereafter called “core and tail length”, in terms of numbers of SNPs) [20]. A core is a string of consecutive SNPs for which phasing is being determined, and the adjacent tails are strings of consecutive SNPs that are adjacent to either end of a core.

A total of *n* phasing analyses with different core and tail lengths were performed, such that each SNP was phased many times as a part of cores that span different SNP windows. Using different lengths of consecutive SNPs addresses the fact that the expected size of shared haplotypes is larger for more closely-related individuals than for less related individuals [21]. When analysing the simulated data, nine different core and tail lengths were considered. Applied combinations of core and tail lengths (core length, tail length) were (150, 200), (200, 200), (250, 100), (250, 200), (300, 100), (300, 200), (350, 50), (350, 100), and (350, 200). All phasing analyses were performed twice considering either offset or non-offset analyses, which resulted in 18 phasing analyses per simulation replicate. Offset analyses were designed to create 50 % overlap between cores of the offset and non-offset analyses, by moving the beginning of each core to halfway along the first core of the non-offset analyses. Because offset and non-offset analyses were always performed together for a specific combination of core and tail lengths, the term “phasing analysis” will hereafter refer to both analyses. Different core lengths combined with offset and non-offset analyses help to remove phasing errors that AlphaPhase1.1 may introduce. For all phasing analyses, 1 % genotype errors and 1 % disagreement between genotypes and haplotypes were allowed. Both the number of surrogate parents across which information pertaining to a phase must be accumulated before this phase can be declared, and the maximum percentage of surrogate disagreements that still allow phase declaration were set to 10. The same settings were used for the real data.

#### Assigning breed origin to haplotypes

A specific haplotype detected in a crossbred animal is fully informative if this haplotype occurs in only one of the purebred populations. Therefore, after each phasing analysis, the next step involved listing all haplotypes that were phased in the purebred populations. Subsequently, haplotypes that were phased within only one purebred population were identified and their origin was assigned to this breed, and added to the library associated with this breed origin. Thus, a library of haplotypes assigned to a specific breed origin included all assigned haplotypes that were derived from all the phasing analyses (i.e., across the different core and tail lengths, as well as across the offset and non-offset analyses).

Allocation of a haplotype to a unique purebred population may not always be possible, especially for closely-related populations, which can share large haplotypes. To allow assignment of breed origin for most haplotypes, a ‘relaxation factor’ (f_r_) was applied. Using this f_r_, a haplotype was assigned to a purebred population, if less than f_r_ % of all copies of that haplotype were observed in the other purebred populations. Haplotypes that did not fulfil this condition remained unassigned. Relaxation factors f_r_ of 0, 10 and 20 % were considered in this study.

#### Assigning breed origin to alleles of crossbred animals

Assignment of breed origin to each SNP allele of a crossbred animal was based on (1) the library of assigned haplotypes, (2) breed composition of the crossbred animal [e.g., BC or A(BC)], and (3) zygosity of the SNP genotype of the crossbred animal (i.e., homozygosity or heterozygosity) to which the considered allele contributes. Breed composition and zygosity of the SNP genotypes were assumed to be correct. A pseudo-code for assigning breed origin to alleles is described in the “Appendix”.

Haplotypes in crossbred animals were traced back in the library of assigned haplotypes, which provided breed origin for each allele of the haplotypes in crossbred animals. Because *n* offset and non-offset phasing analyses were performed (e.g., *n* = 9 and 2*n* = 18 for this study), each allele could receive a maximum of 2*n* possible breed origins. A smaller number of breed origin assignments was also possible for a specific allele if the haplotype of this allele was not phased or not assigned a breed origin in some analyses. If breed origin assignments were not the same for an allele across the different phasing analyses; but in agreement with the breed composition and zygosity of the SNP genotype of the crossbred animal, the most frequent breed assignment was considered as the breed origin.

As mentioned previously, the BOA approach takes breed composition of a crossbred animal into account to assign breed origin. This knowledge helps to assign breed origin to an allele that is present in several haplotypes that are assigned to different breed origins. For the two-way and three-way crossbred animals, one of the two alleles of each SNP must originate from the paternal breed. Assigning the paternal allele first reduces the possibilities of assignment for the maternal allele. For example, for a homozygous SNP for an A(BC) animal, its breed composition (i.e., its paternal breed is A) helps to assign one allele to breed A, even if different haplotypes that contain this allele were assigned different breed origins.

Zygosity of the SNP genotype was also taken into account to avoid disagreement between genotypes based on the input data and based on the two phased haplotypes. Such issues can arise because AlphaPhase1.1 allows disagreements between genotypes and haplotypes. Thus, an allele that is present in a haplotype may differ from the allele observed in the genotype, which results in a heterozygous genotype based on the input data and a homozygous genotype based on the two phased haplotypes (or vice versa). The BOA approach considers as correct the zygosity of the SNP genotype based on the input data.

### Accuracy of allele origin assignment and effects of different settings using simulated data

For each breeding scenario (i.e., closely-related, distantly-related, or unrelated breeds) combined with each value of f_r_, i.e. 0, 10 or 20 %, accuracy of assignment of allele origin was computed for chromosomes chr1 and chr2 separately on a per animal basis. Breed origin assignment was assessed for each BC and A(BC) crossbred animal. The minimum, average, and maximum percentage of alleles of an animal that were assigned a correct or incorrect breed origin (%correct or %incorrect) or that were unassigned (%unknown) were computed. All scenarios were replicated 10 times and %correct, %incorrect, and %unknown were averaged across animals and replicates.

Effects of the number of core and tail lengths and of the number of offset and non-offset phasing analyses considered for assignment of allele origin were studied through forward selection, with the aim to identify useful sets of settings to be used for phasing. Starting with no phasing analysis, addition of each offset and non-offset phasing analysis was tested using the average %correct for A(BC) animals as criterion. Then, the offset and non-offset phasing analysis that improved average %correct most was added. This process was repeated until all offset and non-offset phasing analyses were added. The forward selection was performed for all scenarios and values of f_r_. The order, in which the different phasing analyses were added, was studied to evaluate which (combination of) settings yielded the highest average % of correctly assigned alleles.

### Assignment of allele origin using real data

Assignment of allele origin was performed for all EF and D(EF) crossbred animals by considering the nine offset and non-offset phasing analyses (i.e., a total of 18 phasing analyses) for SSC2 and 18. For each relaxation factor (i.e., 0, 10, and 20 %), the average, minimum and maximum percentages of assigned alleles (%assigned) for each EF and D(EF) animal were computed for each chromosome separately. The percentage of animals with at least 80 % assigned alleles was also computed, as an arbitrary measure to evaluate the number of genotypes that would be useful for subsequent analysis, as well as the average %assigned for each of the breed origins that contributed to the EF or D(EF) animals.

## Results

### Simulated data

#### Characteristics of simulated data

For each replicate of the simulated data, about 1000 purebred animals and 420 crossbred animals were randomly selected from the three-way crossbreeding program to assign breed origin to alleles (Table 1). The two simulated chromosomes had on average 15 SNPs per cM across all replicates and scenarios, i.e., 4811 SNPs for chr1 and 926 SNPs for chr2 (Table 1). MAF of the SNPs in the purebred animals for chr1 and chr2, averaged across SNPs and replicates, ranged from 0.27 to 0.30 for closely-related breeds, from 0.21 to 0.28 for distantly-related breeds, and from 0.15 to 0.25 for unrelated breeds. To quantify the divergence between the simulated breeds, the estimated global Wright’s *F*_ST_, i.e., the average inbreeding rate of the sub-population relative to the whole population, were equal to 0.04 (±0.00) for the closely-related breeds, 0.13 (±0.01) for the distantly-related breeds, and 0.28 (±0.02) for the unrelated breeds (Table 1) [22].

#### Percentage of assigned alleles

In most cases, less than 5 % of the alleles observed in the crossbred animals were not (correctly or incorrectly) assigned breed origin (Table 2; Additional file 1: Tables S1, S2, S3, S4). The highest average percentage of unassigned alleles per animal (average %unknown) was equal to 10.8 % (±1.5 %), and was found for chr2 of A(BC) animals from closely-related breeds and with an f_r_ of 0 % (Table 2). For both chromosomes, the average %unknown was close to 0 % when BC and A(BC) animals were from unrelated breeds and with an f_r_ of 20 % (Table 2; Additional file 1: Tables S1, S2, S3, S4). These low %unknown (or, equivalently, high percentages of allele assignments) were substantiated by the observation that all BC and A(BC) animals from distantly-related and unrelated breeds had at least 80 % of their alleles assigned for chr1. More than 97 % of these animals had also 80 % of their alleles assigned for chr2. As the distance between breeds decreased, the percentage of animals having at least 80 % of alleles assigned decreased (e.g., between 80.9 and 93.3 % for chr2 for A(BC) animals from closely-related breeds). All these results show that the average %unknown decreased as the distance between breeds or f_r_ increased. The average %unknown was also affected by characteristics of the chromosome such as length or number of SNPs present on the chromosome (SNP densities were similar for chr1 and chr2).

**Table 2:** Percentages of alleles correctly (%correct) or incorrectly (%incorrect) assigned a breed origin or unassigned (%unknown) for a crossbred animal, and percentages of crossbred animals having at least 80 % assigned alleles using simulated data

Scenarios	f_r_^a^	%correct		%incorrect		%unknown		>80 %assigned
		Average	Max	Average	Max	Average	Max	
Chromosome 1 of BC animals								
Closely-related breeds	20	95.15 (0.37)	99.99 (0.03)	0.39 (0.02)	2.99 (0.86)	4.46 (0.37)	22.38 (5.20)	99.76 (0.22)
	0	92.42 (0.57)	99.92 (0.12)	0.36 (0.02)	2.49 (0.36)	7.22 (0.56)	26.69 (4.16)	98.43 (0.93)
Distantly-related breeds	20	98.09 (0.09)	100.00 (0.00)	0.32 (0.02)	2.12 (0.43)	1.60 (0.08)	9.56 (1.12)	100.00 (0.00)
	0	97.92 (0.22)	100.00 (0.00)	0.30 (0.02)	2.05 (0.46)	1.77 (0.21)	10.30 (1.27)	100.00 (0.00)
Unrelated-breeds	20	98.58 (0.13)	100.00 (0.00)	0.28 (0.04)	1.59 (0.32)	1.14 (0.11)	7.56 (1.36)	100.00 (0.00)
	0	98.58 (0.14)	100.00 (0.00)	0.28 (0.04)	1.59 (0.32)	1.14 (0.11)	7.56 (1.36)	100.00 (0.00)
Chromosome 2 of BC animals								
Closely-related breeds	20	94.05 (0.71)	100.00 (0.00)	0.47 (0.07)	8.90 (3.33)	5.48 (0.68)	53.47 (8.84)	93.33 (2.19)
	0	90.72 (1.32)	100.00 (0.00)	0.45 (0.05)	8.91 (2.85)	8.83 (1.31)	66.96 (15.52)	85.00 (2.75)
Distantly-related breeds	20	97.62 (0.39)	100.00 (0.00)	0.38 (0.06)	6.60 (1.68)	2.00 (0.35)	26.16 (5.80)	99.32 (0.70)
	0	97.44 (0.39)	100.00 (0.00)	0.37 (0.06)	6.33 (1.83)	2.19 (0.35)	29.22 (5.91)	98.94 (0.73)
Unrelated breeds	20	98.00 (0.48)	100.00 (0.00)	0.36 (0.11)	4.25 (1.07)	1.64 (0.42)	26.03 (10.98)	99.59 (0.53)
	0	98.00 (0.49)	100.00 (0.00)	0.35 (0.11)	3.95 (0.74)	1.65 (0.43)	26.03 (10.98)	99.57 (0.56)
Chromosome 1 of A(BC) animals								
Closely-related breeds	20	93.18 (0.43)	99.77 (0.21)	1.69 (0.10)	8.96 (1.56)	5.13 (0.41)	19.71 (3.23)	99.81 (0.24)
	0	88.91 (0.63)	99.18 (0.58)	1.58 (0.10)	8.47 (1.33)	9.51 (0.59)	31.48 (3.78)	96.14 (1.02)
Distantly-related breeds	20	96.21 (0.16)	99.93 (0.06)	0.96 (0.09)	4.98 (0.84)	2.83 (0.11)	11.46 (1.07)	100.00 (0.00)
	0	95.92 (0.23)	99.92 (0.05)	0.95 (0.10)	4.78 (0.67)	3.13 (0.18)	12.09 (0.96)	100.00 (0.00)
Unrelated breeds	20	96.99 (0.15)	99.97 (0.03)	0.68 (0.04)	3.19 (0.72)	2.33 (0.14)	9.99 (1.83)	100.00 (0.00)
	0	96.97 (0.15)	99.97 (0.03)	0.69 (0.04)	3.21 (0.70)	2.34 (0.14)	10.14 (1.69)	100.00 (0.00)
Chromosome 2 of A(BC) animals								
Closely-related breeds	20	91.64 (0.95)	100.00 (0.00)	1.99 (0.17)	27.62 (6.67)	6.37 (0.91)	54.24 (10.13)	92.45 (2.49)
	0	87.37 (1.55)	100.00 (0.00)	1.83 (0.17)	24.18 (4.61)	10.80 (1.47)	66.40 (9.10)	80.89 (4.28)
Distantly-related breeds	20	95.51 (0.50)	100.00 (0.00)	1.14 (0.15)	15.66 (4.72)	3.35 (0.42)	32.28 (8.57)	98.57 (1.08)
	0	95.11 (0.48)	100.00 (0.00)	1.09 (0.15)	16.16 (4.55)	3.79 (0.39)	33.51 (8.69)	97.94 (1.06)
Unrelated breeds	20	95.83 (0.65)	100.00 (0.00)	0.87 (0.14)	11.28 (1.77)	3.30 (0.59)	32.24 (11.40)	98.04 (1.16)
	0	95.78 (0.66)	100.00 (0.00)	0.87 (0.14)	11.58 (1.62)	3.35 (0.60)	32.83 (11.53)	98.00 (1.19)

Results are averages (SD) across the 10 replicates

^a^f_r_ = relaxation factor

While most of the animals had only a few unassigned alleles, %unknown reached high values for some animals, especially for chr2. For example, the maximum %unknown for chr2 of a BC animal from closely-related breeds was equal to 67.0 % (±15.5 %) (Table 2; Additional file 1: Tables S1, S2, S3, S4). Therefore, for some BC or A(BC) animals, breed origin was not assigned to many of their alleles.

#### Accuracy of allele assignment

Across all analyses, the %incorrect averaged across animals and across replicates was at most equal to 1.99 % (±0.17 %) for chr2 of A(BC) animals from closely-related breeds with an f_r_ of 20 %. The %incorrect decreased slightly as the distance between breeds increased, or f_r_ decreased (Table 2; Additional file 1: Tables S1, S2, S3, S4). Characteristics of the chromosome, such as length and number of SNPs, also influenced %incorrect. For all scenarios and values of f_r_, %incorrect was always higher for chr2 than for chr1 but it was not possible to determine if this was due to the length of the chromosome or the number of SNPs. In addition to the average %incorrect, knowing the maximum %incorrect for an animal may be important. For both BC and A(BC) animals, the highest %incorrect was obtained for chr2 for the closely-related breed scenario (Table 2; Additional file 1: Tables S1, S2, S3, S4). The highest maximum %incorrect (averaged across all replicates) reached 10.1 % for a BC animal (f_r_ = 10 %) and 27.6 % for an A(BC) animal (f_r_ = 20 %).

Regardless of the scenario, the average %incorrect was similar and low (i.e., always less than 2.0 % for all scenarios, f_r_ values, both chromosomes, and all animals). Since the average %incorrect remained relatively constant, the effect of the different factors on the average %correct was the inverse of that on the average %unknown. The average %correct was affected by the characteristics of the chromosome and increased as the distance between breeds or f_r_ increased. For all scenarios, f_r_ values and both chromosomes, the average %correct ranged from 90.7 to 98.6 % for BC animals and from 87.4 to 97.0 % for A(BC) animals. Some BC and A(BC) animals had (close to) 100 % of alleles with correctly assigned breed origins (Table 2; Additional file 1: Tables S1, S2, S3, S4). Comparing the results for %incorrect and %unassigned showed that the BOA approach was more likely to consider the origin of an allele as unknown than to assign an incorrect breed origin.

#### Impact of distance between breeds

A greater distance between breeds had a favourable effect on the percentage and accuracy of breed origin assignment, while this relationship appears to reach a plateau at distances greater than 20 generations. Results for distantly-related and unrelated breeds were similar regardless of the chromosome, f_r_, or type of crossbred animals. Increasing the distance between breeds from 20 (*F*_ST_ = 0.13; Table 1) to 50 generations (*F*_ST_ = 0.28; Table 1) had less impact on allele assignment than increasing it from 5 (*F*_ST_ = 0.05; Table 1) to 20 generations (i.e., between closely- and distantly-related breeds).

#### Impact of the relaxation factor

The relaxation factor f_r_ was introduced because many haplotypes can be present in more than one purebred population, especially for closely-related populations that can share long haplotypes. Indeed, the impact of f_r_ was greater for closely-related breeds than for distantly-related breeds. For example, the largest increase of the average %correct due to increasing f_r_ from 0 to 20 % was equal to 4.27 %, for chr2 of A(BC) animals for closely-related breeds (Table 2). Increasing f_r_ from 0 to 20 % also increased the percentages of BC and A(BC) animals having at least 80 % of alleles assigned. The largest increase was observed for chr2 for both BC animals (i.e., an increase of 8.3 %) and A(BC) animals (i.e., an increase of 11.6 %) from closely-related breeds (Table 2; Additional file 1: Tables S1, S2, S3, S4). Increasing f_r_ did not or only slightly affect the average %incorrect; the largest increase, 0.16 %, was observed for chr2 of A(BC) animals from closely-related breeds (Table 2). Given that the average %incorrect remained almost constant, the effect of increasing f_r_ mainly resulted in a greater percentage of correctly assigned alleles that previously fell in the unknown origin category.

#### Impact of core and tail lengths

The effect of choosing specific core and tail lengths was analysed by calculating Spearman rank correlations (r_s_) between the order of the phasing analyses obtained from the forward selection and a predefined order of the same phasing analyses. The predefined ranking ordered the phasing analyses according to decreasing core and tail lengths. If two different combinations of core and tail lengths had the same total length, the predefined ranking followed a decreasing core length. Analyses with longer core and tail lengths are preferred because they have smaller computational requirements. High and positive r_s_ indicate that long core and tail lengths are preferred to maximize the average %correct. More details on r_s_ with averages and SD across all replicates are in Table 3; Additional file 1: Table S5.

**Table 3:** Spearman rank correlations between the order of the phasing analyses obtained from forward selection and a predefined order for simulated data

Scenarios	Relaxation factor	Chromosome 1	Chromosome 2
Closely-related breeds	20	0.41 (0.09)	0.09 (0.17)
	0	0.56 (0.19)	0.30 (0.14)
Distantly-related breeds	20	0.05 (0.16)	−0.27 (0.20)
	0	0.29 (0.13)	−0.04 (0.22)
Unrelated breeds	20	−0.22 (0.08)	−0.13 (0.23)
	0	−0.21 (0.11)	−0.15 (0.23)

Reported results are averages (SD) across all replicates

**Fig. 1:**
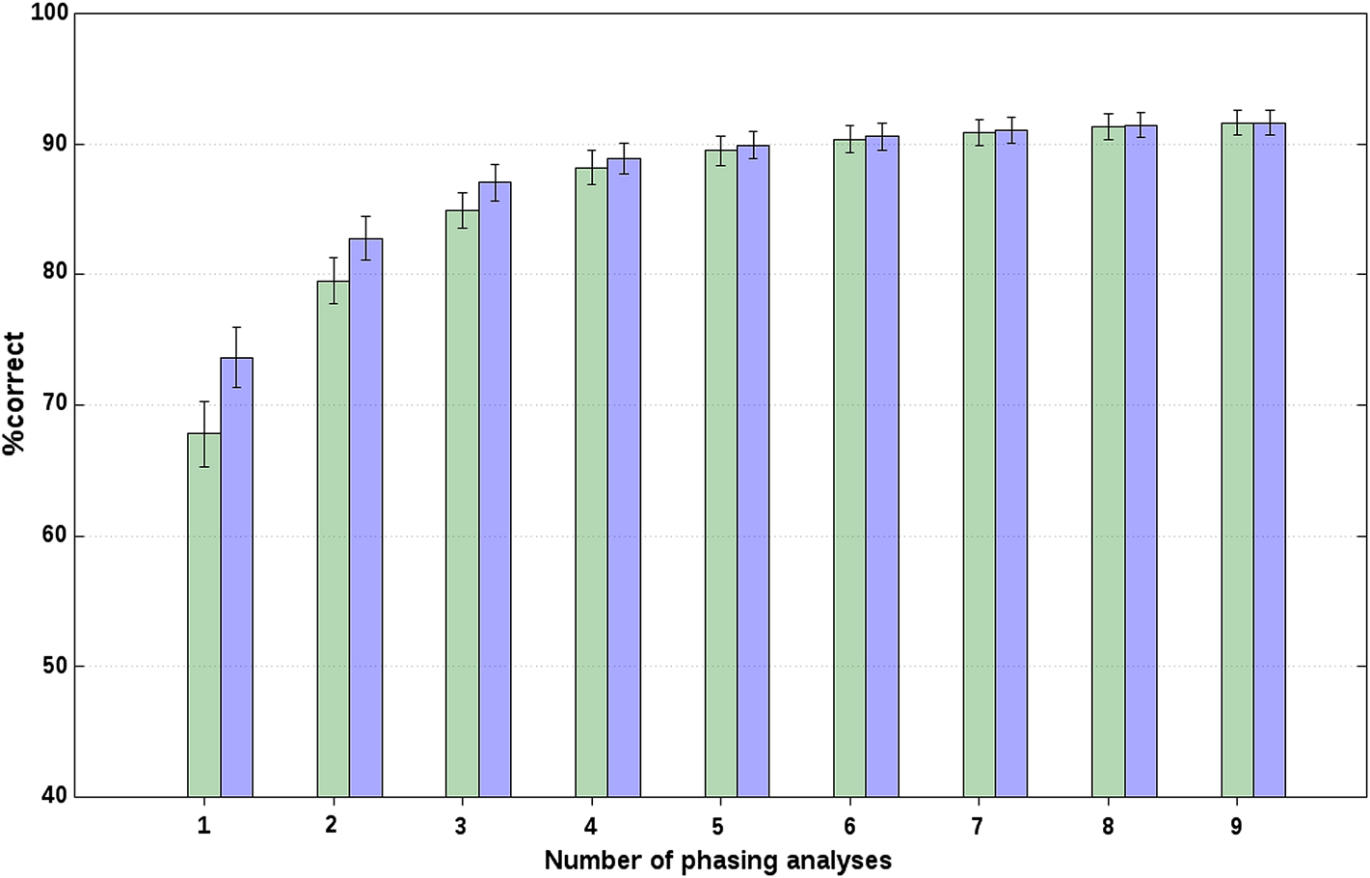
Percentages of correct allelic assignments with a relaxation factor of 20 % for simulated closely-related breed data. Minimum (*green*) and maximum (*blue*) percentages of correct allelic assignments, for SSC2 and averaged across A(BC) animals of closely-related breeds, as a function of the number of offset and non-offset phasing analyses that were selected based on a forward selection. Reported results are averages and SD across all replicates

**Fig. 2:**
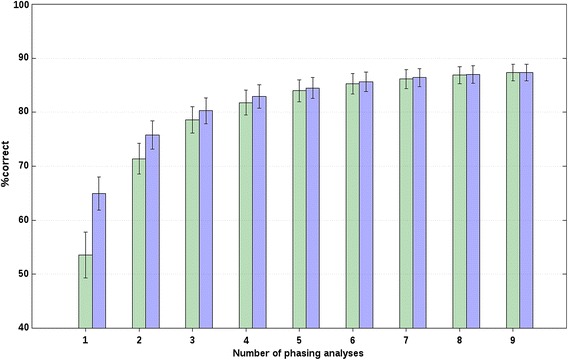
Percentages of correct allelic assignments with a relaxation factor of 0 % for simulated closely-related breed data. Minimum (*green*) and maximum (*blue*) percentages of correct allelic assignments, for SSC2 and averaged across A(BC) animals of closely-related breeds, as a function of the number of offset and non-offset phasing analyses that were selected based on a forward selection. Reported results are averages and SD across all replicates

**Fig. 3:**
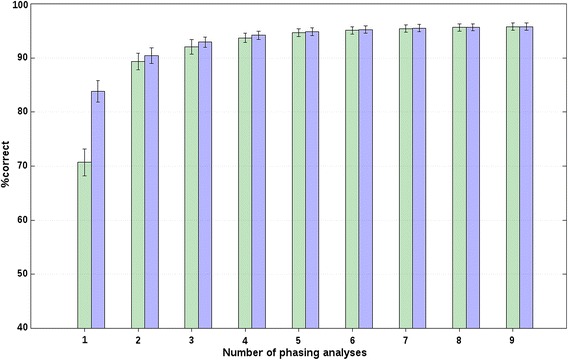
Percentages of correct allelic assignments with a relaxation factor of 0 % for simulated unrelated breed data. Minimum (*green*) and maximum (*blue*) percentages of correct allelic assignments, for SSC2 and averaged across A(BC) animals of unrelated breeds, as a function of the number of offset and non-offset phasing analyses that were selected based on a forward selection. Reported results are averages and SD across all replicates

For both chromosomes, r_s_ decreased with increasing distance between breeds (or, with increasing *F*_ST_), which suggests that longer core and tail lengths should be used to analyse data from closely-related breeds compared to unrelated breeds, as expected. Also, for all scenarios, f_r_, and both chromosomes, we evaluated the effect of adding additional phasing analyses on %correct based on the forward selection. The %correct followed a logarithmic trend that rapidly increased for about the first four analyses, as shown in Figs. 1 and 2 for chr2 of A(BC) animals from closely-related breeds with an f_r_ of 20 and 0 %, respectively, and in Fig. 3 for chr2 of A(BC) animals from unrelated breeds with an f_r_ of 0 %. Figures S1, S2 and S3 show the results obtained for the other scenarios and f_r_ (see Additional file 2: Figures S1, S2, S3). These figures show the minimum and maximum %correct averaged across all A(BC) animals and all replicates that were obtained when one additional phasing analysis was considered by the BOA approach. Relatively large average differences between the maximum and minimum average %correct were observed when only one phasing analysis was considered. For example, for chr2 of A(BC) animals from closely-related breeds, the average difference between the minimum and maximum average %correct that was obtained when only one phasing analysis was considered, was between 5.1 (f_r_ = 20 %; Fig. 1) and 14.4 % (f_r_ = 0 %; Fig. 2). Similar results were obtained for all other scenarios (e.g., Fig. 3; Additional file 2: Figures S1, S2, S3). Thus, these results show that the choice of core and tail lengths has an impact on %correct. However, based on these results and the estimates of r_s_ with their large associated SD, it does not seem possible to provide precise indications on the best core and tail lengths, since they were quite specific to the data analysed.

### Real data

#### Characteristics of the real data

Genotypes for SSC2 and 18 of about 950 D purebred animals and of at least 1800 E and F purebred animals were available. Genotypes for 324 EF animals and for 241 D(EF) animals were also available (Table 1). SSC2 and 18 included 2496 and 1129 SNPs, respectively. The estimated global *F*_ST_ was equal to 0.15 (Table 1).

#### Percentage of assigned alleles

For SSC2 of the EF animals, the average %assigned ranged from 89.0 (f_r_ = 0 %) to 92.5 % (f_r_ = 20 %). The minimum %assigned ranged from 40.1 (f_r_ = 0 %) to 34.9 % (f_r_ = 20 %). Between 87.0 (f_r_ = 0 %) and 93.8 % (f_r_ = 20 %) of the EF animals had at least 80 % of their alleles on SSC2 assigned (Table 4; Additional file 1: Table S6). For SSC18 of the same EF animals, the average %assigned ranged from 88.8 (f_r_ = 0 %) to 90.4 % (f_r_ = 20 %) and between 81.2 (f_r_ = 0 %) and 85.2 % (f_r_ = 20 %) of the EF animals had at least 80 % of their alleles on SSC18 assigned (Table 4). Similar percentages were observed for D(EF) animals (Table 4; Additional file 1: Table S6). As in the simulated data, the average %assigned and percentages of animals with at least 80 % of assigned alleles increased with increasing f_r_.

**Table 4:** Percentages of assigned alleles on SSC2 and SSC18 for an EF or a D(EF) animal and percentages of EF and D(EF) animals with at least 80 % assigned alleles

Chromosome	f_r_^a^	%assigned				Percentage of animals with more than 80 % assigned
		Average	SD	Min	Max	
SSC2						
EF	20	92.45	8.14	34.86	100.00	93.83
	0	88.90	9.19	40.07	100.00	87.03
D(EF)	20	92.54	8.51	44.81	99.98	96.68
	0	89.58	9.50	44.62	99.92	88.38
SSC18						
EF	20	90.35	11.07	26.44	100.00	85.19
	0	88.77	11.70	19.84	100.00	81.17
D(EF)	20	90.06	11.17	45.22	100.00	82.98
	0	88.57	11.83	44.95	100.00	79.67

^a^f_r_ = relaxation factor

#### Breed origin of alleles

**Fig. 4:**
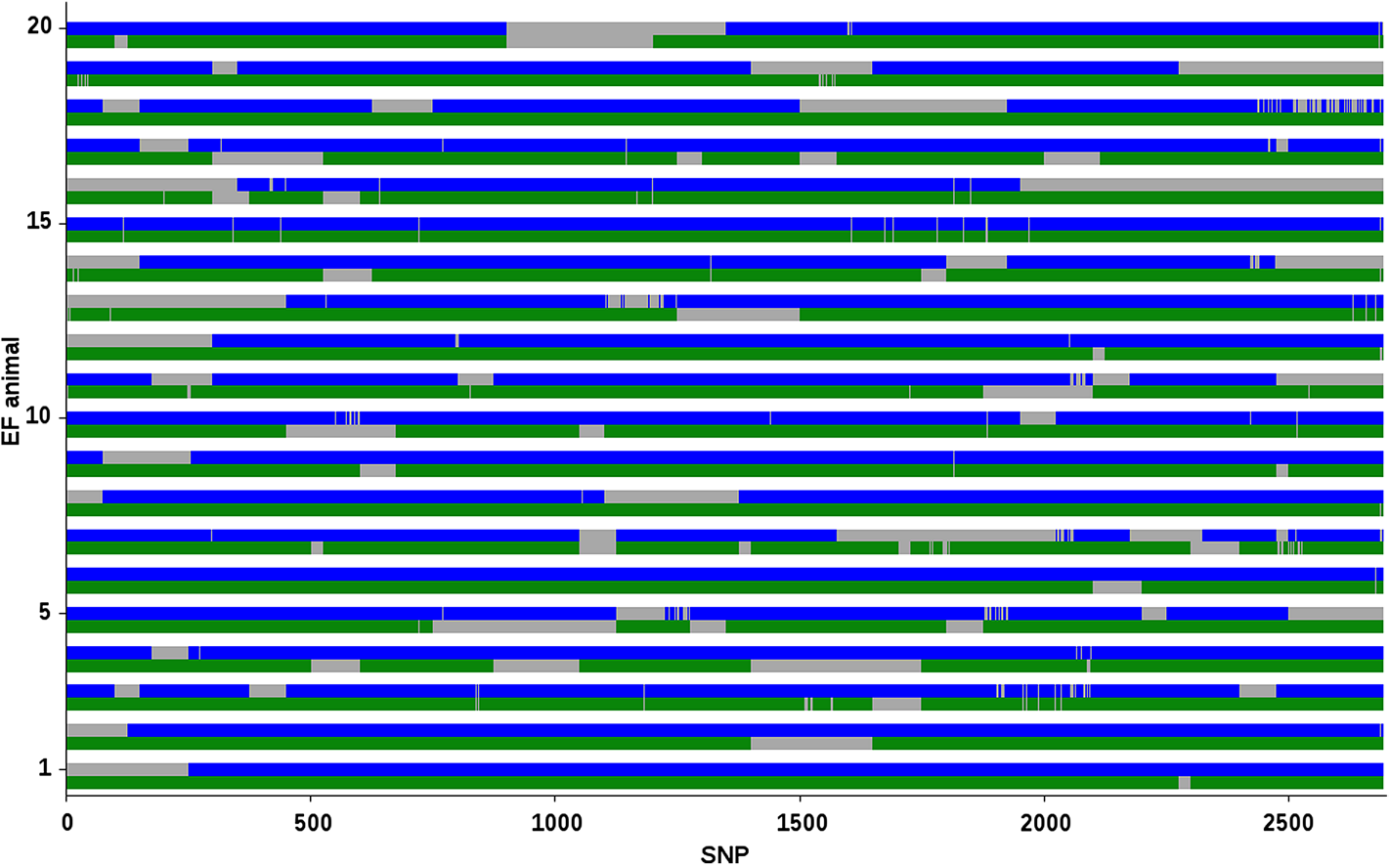
Breed origin of the two alleles for each SNP on SSC2 in 20 EF animals. Alleles from breeds E and F are in *green* and *blue*, respectively. *Grey regions* are unassigned alleles. Results are obtained from the proposed algorithm with a relaxation factor of 0 %

**Fig. 5:**
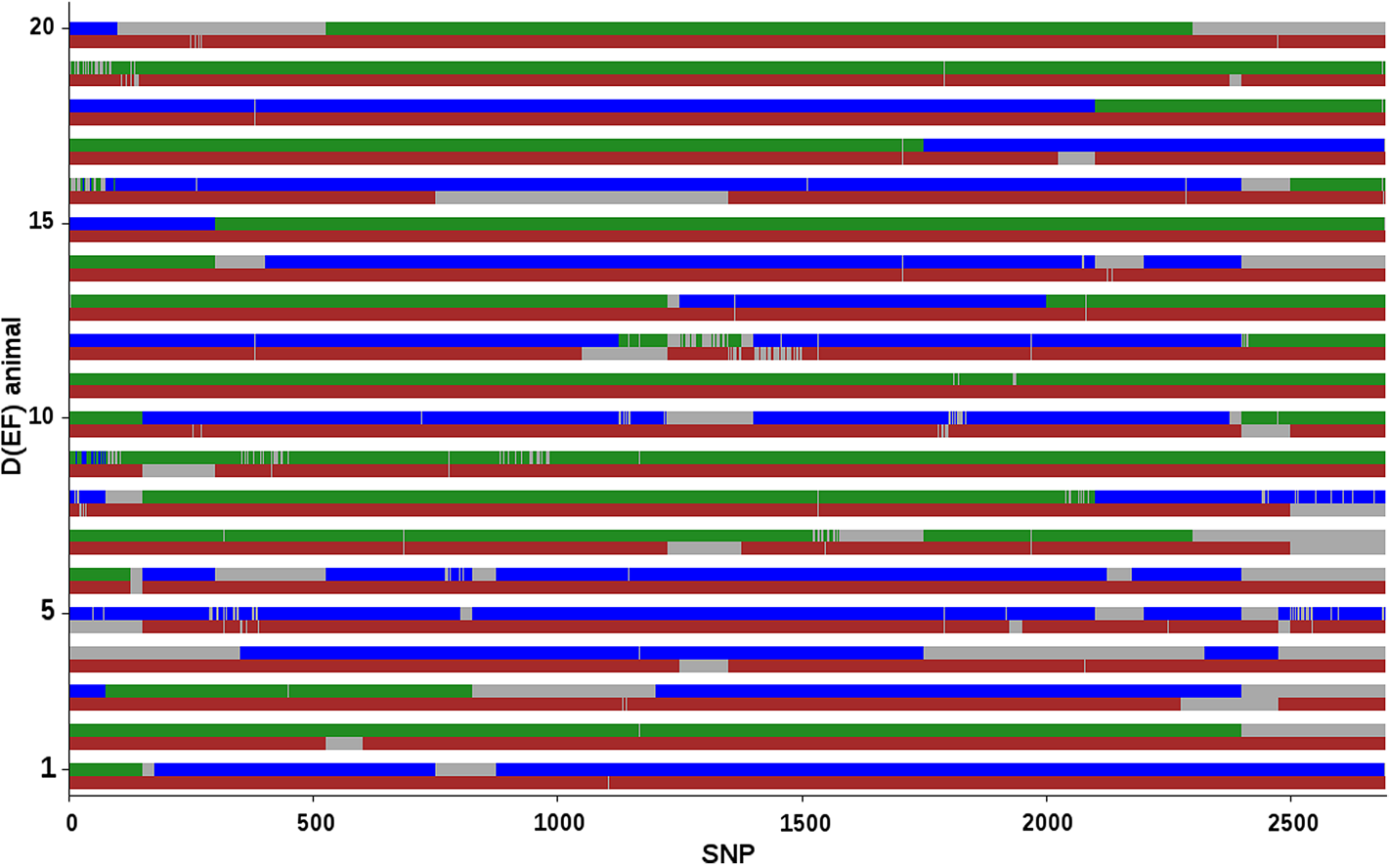
Breed origin of the two alleles for each SNP on SSC2 in 20 D(EF) animals. Alleles from breeds D, E, and F are in *brown*, *green*, and *blue*, respectively. *Grey regions* are unassigned alleles. Results are obtained from the proposed algorithm with a relaxation factor of 0 %

Because correctness of breed origin assignment could not be assessed for real data, average %assigned relative to breed origin for an EF or a D(EF) animal are reported in Table 5; Additional file 1: Table S7. Figures 4 and 5 show breed origins assigned to alleles across SSC2 for 20 randomly selected EF and D(EF) animals, respectively. The results were consistent with our expectations for both SSC2 and 18. Average percentages for EF animals were close to 50 % for breeds E and F. The lower percentages obtained for breed F (e.g., 44.2 % for SSC2 and f_r_ = 20 %) can be attributed to the fact that the BOA approach preferably assigns breed origin of an allele as unknown rather than a possible incorrect origin. For example, some alleles of EF animal 1 (Fig. 4) were unassigned, while the corresponding alleles at the same SNPs were assigned an E breed origin. For both chromosomes of D(EF) animals, the average %assigned was close to 50 % for breed D (i.e. the sire breed), and close to 25 % for breeds E and F (i.e., the maternal breeds;), as expected (Table 5; Additional file 1: Table S7), and Fig. 5).

**Table 5:** Average (SD) percentages of alleles on SSC2 and SSC18 assigned to each parental breed for an EF or a D(EF) animal

Animals	Breed D	Breed E	Breed F
EF			
SSC2	–	48.24 (4.31)	44.21 (6.35)
SSC18	–	47.17 (6.94)	43.18 (8.53)
D(EF)			
SSC2	47.16 (7.15)	23.91 (17.70)	21.47 (17.00)
SSC18	47.51 (7.67)	21.72 (19.17)	20.83 (17.95)

Relaxation factor was equal to 20 %

Some maternal chromosomes of D(EF) animals were (mainly) assigned to one of the two maternal breed origins (e.g., animals 5, 9, or 19; Fig. 5). These maternal chromosomes show a limited number of recombinations, as expected, and the percentages of breed origin for individual chromosomes can deviate considerably from their expectation (i.e., from 25 %). In addition, we found that recombinations occurred more frequently towards the end of the chromosomes and less in the middle based on the physical map, which is consistent with the genetic map length and recombination rate being higher in the more distal part of the chromosome [23].

#### Impact of core and tail lengths

**Fig. 6:**
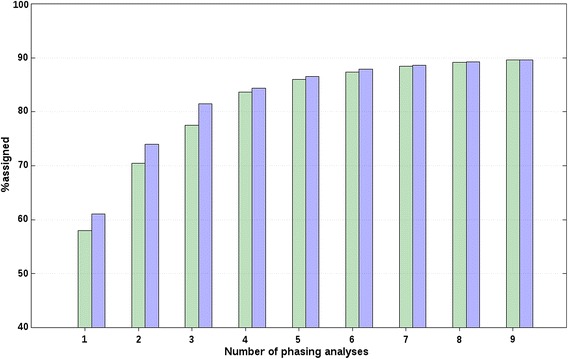
Percentages of allelic assignments for D(EF) animals with a relaxation factor of 0 %. Minimum (*green*) and maximum (*blue*) percentages of allelic assignments for SSC2, as a function of the number of phasing analyses that were selected based on a forward selection

**Fig. 7:**
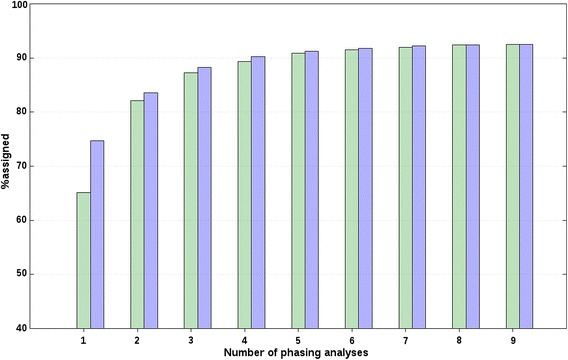
Percentages of allelic assignments for D(EF) animals with a relaxation factor of 20 %. Minimum (*green*) and maximum (*blue*) percentages of allelic assignments for SSC2, as a function of the number of phasing analyses that were selected based on a forward selection

The criterion for the forward selection for D(EF) animals was the %assigned, instead of the average %correct, because it was not possible to determine the correctness of allele assignments. For SSC2, rank correlations between the order of the phasing analyses obtained from the forward selection and the predefined order of the same phasing analyses were −0.02 for f_r_ = 0 and 10 % and −0.03 for f_r_ = 20 %. For SSC18, rank correlations were between −0.63 for f_r_ = 0 % and −0.32 for f_r_ = 20 %. The effect of adding phasing analyses based on the forward selection for SSC2 is presented in Fig. 6 for f_r_ = 0 %, and in Fig. 7 for f_r_ = 20 %; similar trends were observed for SSC18. Thus, similar to the results obtained with the simulated data, the effect of considering one additional phasing analysis at a time by the BOA approach followed a logarithmic trend that levelled out after combining four analyses.

## Discussion

The objectives of this study were (1) to develop an approach (BOA) for assigning breed origin to alleles of crossbred animals, and (2) to study its accuracy as a function of different factors. The results obtained from simulated and real data showed that the BOA approach accurately assigns breed origin to alleles of crossbred animals, and that its accuracy depends on various factors, such as the distance between the parental breeds.

### Distance between breeds

The global Wright’s *F*_ST_ statistic measures the average inbreeding in a sub-population relative to the whole population and takes the effect of population subdivision into account. For example, for the distantly-related breeds, the estimated global *F*_ST_ was equal to 0.13, which indicated that about 13 % of the genetic variance in the combined population can be attributed to differentiation of the breeds (Table 1) [22, 24]. Based on the estimated global *F*_ST_, the distances among the three breeds included in the real dataset were similar to those between the distantly-related breeds included in the simulated data. Comparison of results for assignment of breed origin showed that the %assigned was slightly lower for the real data than expected based on results obtained with the simulated data for distantly-related breeds. This may be explained by breed composition errors, genotype errors, or the structure of the purebred populations that were included in the real dataset.

### The BOA approach and additional rules

The BOA approach assigned a breed origin to each allele of each SNP, based on a library of assigned haplotypes, the zygosity of the SNP genotype to which the considered allele contributes, and breed composition of the crossbred animal, if a most frequent breed assignment was observed. The BOA approach was specifically designed to determine breed origin of alleles in crossbred animals from well-defined crossbreeding schemes, and for which the purebred populations are up to two generations back. Breed composition of the crossbred animals, or at least its expectation, is expected to be known. In addition, the BOA approach was able to deal with scenarios that involved closely-related breeds. These characteristics can be overlooked by software tools that were developed to infer local ancestry in (recently) admixed populations [6–9], in which admixed individuals are mated to produce the next generation and, these tools may, therefore, not be adequate for the crossbreeding situation. As for the BOA approach, most of these tools require phased genotypes for the ancestral populations (e.g., [7–9]) and inference of local ancestry is mainly realized through a Markov process (e.g., [6–9]). These methods also use allele frequencies, levels of LD between subsets of SNPs in the ancestral populations, pedigree information, and/or recombination rates. While such information is not (directly) used by the BOA approach, it could be useful to increase %assigned. Some additional rules to the BOA approach, e.g. based on allele frequencies, and their effects on allele assignments, are discussed below. Nevertheless, while these software tools may not be adequate for the typical crossbreeding programs for pigs or chicken, they may be useful for other livestock production systems, such as those for cattle, for which crossbreeding schemes are more complex [2]. It should be possible, however, to adapt the BOA approach for these more complex scenarios.

Across the simulated scenarios, the percentage of alleles in a three-way crossbred animal that were correctly assigned to breed origin was higher than 90 %, and the percentage of incorrectly assigned alleles was always lower than 2 %. For the remaining alleles, between 0 and 10 % of all alleles in a three-way crossbred animal had no breed origin assigned. Additional rules to the BOA approach, which could be applied post-processing, could increase the %assigned. For example, assignment of the other allele at the SNP and assignments of alleles at other SNPs near the unassigned allele were not considered by the BOA approach. If one allele at a SNP was not present in at least one assigned haplotype, breed origin was not assigned to this allele, even if breed origin was assigned to the other allele at this SNP. This explains why for some SNP genotypes, breed origin was assigned for one but not the other allele, even for crossbred animals that originated from only two breeds (e.g., animal 1 in Fig. 4).The reason why breed origin of an allele was not assigned based on the assignment of the other allele was to avoid adding incorrect assignments in case the first allele was incorrectly assigned, which could increase the average %assigned but also the %incorrect. For the same reason, breed origins of assigned alleles in the neighbourhood of unassigned alleles were not used to assign breed origin to these unassigned alleles.

To test the accuracy of allele assignments by using information of assigned alleles, additional rules were added as a post-processing step of the BOA approach in order to assign breed origin to (1) the paternal allele if the maternal allele was already assigned, (2) the maternal allele if the paternal allele was assigned and if the considered animal originated from only two breeds, and (3) alleles if they were surrounded by alleles that were assigned the same breed origin, and if these two assigned alleles were present in the same haplotype that had the smallest core length and that was assigned this breed origin. Surrounding assigned alleles may be separated by several unassigned alleles from a considered unassigned allele. Pseudo-code for these additional rules can be found in the “Appendix”. The additional rules were applied to both simulated and real data (results not shown), and increased the number of assigned alleles by increasing both the %correct and %incorrect. For example, for chr2 of A(BC) animals from closely-related breeds (f_r_ = 0 % and nine phasing analyses), the average %correct increased by 3.7 % and the average %incorrect increased by 0.2 %. For BC animals of the same scenarios, the average %correct and %incorrect increased by 7.6 and 0.4 %, respectively. The average %correct for BC animals was therefore close to 100 %. Detailed results for chr2 with nine phasing analyses are in Additional file 1: Table S8. While the additional rules increased the number of incorrect assignments (slightly), the impact on average %incorrect, relative to not using the additional rules, decreased as the distance between breeds increased. For the real data, the additional rules assigned breed origin to 93.2 % (i.e., an increase of 2.7 %) of the alleles for D(EF) animals and to 98.4 % (i.e., an increase of 9.5 %) of the alleles on SCC2 for the EF animals, with f_r_ = 0 %. Additional file 2: Figures S4 and S5 show breed origins assigned to alleles along SSC2 for 20 randomly-selected EF and D(EF) animals, respectively. The additional rules were especially beneficial for two-way crossbred animals, which was expected because both paternal and maternal alleles of two-way crossbred animals can be assigned by these rules, but only the paternal alleles of three-way crossbred animals. Furthermore, the greater %assigned was mainly due to the assignment of unassigned second alleles, which was also as expected because haplotypes with a small core length can potentially be shared by several breeds, which limits their assignment of breed origin, and therefore, the increase in %assigned. Because the increase in incorrect assignments was limited, the additional rules should be used in order to maximize the number of alleles for which breed origin is assigned.

The MAF of SNPs in the purebred populations can also provide additional information for assigning breed origin to alleles of crossbred animals. For two-way crossbred animals, incorrect assignments can only be obtained for heterozygous genotypes. Let $q_{B}$ and $q_{C}$ be the MAF at a specific SNP for breeds B and C, respectively, and the minor allele be coded as 0. Without other knowledge (e.g., phased haplotypes), we could assign the same breed origin to the two alleles of an observed heterozygous genotype as the breed origin of the two alleles of the highest expected heterozygous genotype [i.e., the heterozygous genotype with the expected frequency equal to ${\text{max((}}1 - q_{B} )q_{C} ,(1 - q_{C} )q_{B} )$]. Such an assignment would result in all 0 and 1 alleles of the observed heterozygous genotypes for a specific SNP having the same origin across all crossbred animals. The expected %correct for heterozygous genotypes at a specific SNP of a two-way crossbred animal is equal to:

$$\frac{{{ \hbox{max} }\left( {\left( {1 - q_{B} } \right)q_{C} ,\left( {1 - q_{C} } \right)q_{B} } \right)}}{{\left( {1 - q_{B} } \right)q_{C} + \left( {1 - q_{C} } \right)q_{B} }} \times 100.$$

**Fig. 8:**
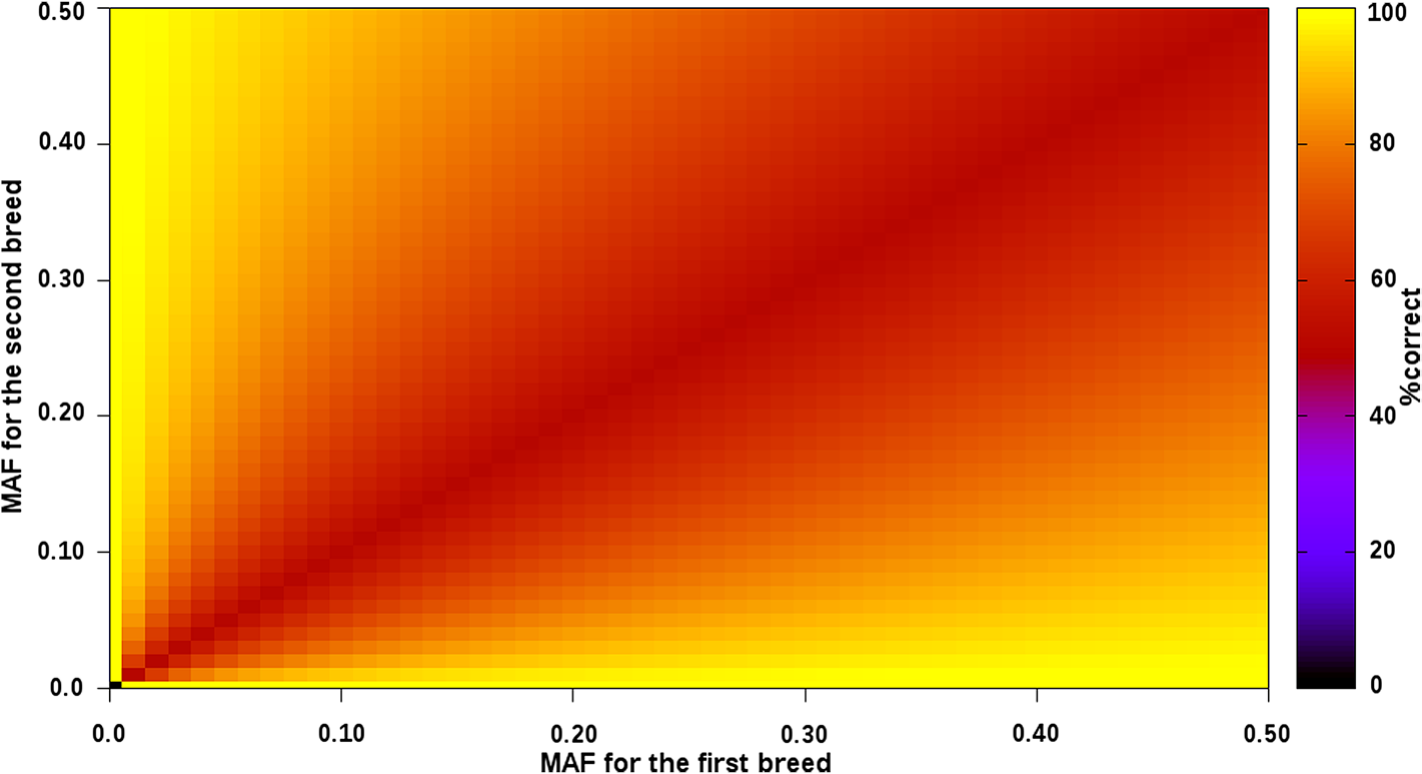
Expected percentages of correct allelic assignments for heterozygous genotypes of two-way crossbred animals. Assignments were based only on minor allelic frequencies (MAF) of the two breeds

If $q_{B} = q_{C}$, the expected %correct is equal to 50 %. If one of the alleles is fixed in one of the two breeds (e.g., $q_{B} = 0$), the expected %correct is equal to 100 %. Figure 8 shows the expected %correct for MAF ranging from 0.00 to 0.50. By applying the BOA approach and the additional rules, more than 97 % of the alleles that are present in heterozygous SNP genotypes were correctly assigned for chr1 of BC animals (f_r_ = 20 %, nine phasing analyses, and averaged across all replicates; Table 6). These results led to an average improvement of at least 26 % over the expected %correct based only on the MAF within the breeds. However, negative differences between observed %correct and expected %correct were observed, which indicates that the BOA approach incorrectly assigned more alleles of observed heterozygous genotypes for a specific SNP than when all alleles were assigned based on MAF. Maximum negative differences ranged from −3.8 % for closely-related breeds to −22.6 % for unrelated breeds. These negative differences were always found when at least one of the alleles was nearly fixed in one of the two breeds, i.e., with a MAF close to 0. Therefore, to increase the accuracy of allele assignment with the BOA approach, MAF should be considered when it is close to 0 for, at least one of the breeds. MAF could also be helpful for observed heterozygous genotypes for which neither of the two alleles is assigned, and could help to assign all alleles of an animal.

**Table 6:** Percentages (averaged across SNPs) of correctly assigned heterozygous genotypes (%correct) for chromosome 1 for BC animals, and differences (diff) between observed and expected %correct for the simulated data

Breeds	%correct	Average diff	Minimum diff	Maximum diff
Closely-related	97.90 (0.14)	35.36 (0.71)	−3.83 (2.85)	49.85 (0.19)
Distantly-related	98.39 (0.11)	29.79 (0.55)	−17.57 (12.35)	49.96 (0.07)
Unrelated	98.31 (0.31)	26.52 (0.83)	−22.62 (17.23)	49.91 (0.12)

Nine phasing analyses and a relaxation factor of 20 % were considered. Results are averages (SD) across the 10 replicates

It is also worth noting that the BOA approach only considers two- and three-way crossbreeding schemes. An extension to a four-way crossbreeding scheme is straightforward by modifying BOA for the paternal allele by applying rules similar to those for the maternal allele. Lower rates of assignment could be expected for four-way crossbred animals, especially because the additional rules that are proposed above cannot be applied for both their paternal and maternal alleles.

### Relaxation factor

The number of haplotypes that segregate only within one of the parental purebred populations may be limited, especially for closely-related breeds which can share many haplotypes. Also, some alleles may be incorrectly phased or not phased at all [20]. For this reason, f_r_ was introduced to allow haplotypes to be assigned, even if a percentage of their copies was observed in other parental purebred populations. Higher f_r_ than the values used here should be avoided because they may increase the %incorrect considerably. For example, f_r_ = 50 % would allow the assignment of breed origin to a haplotype even if 50 % of its copies were observed in the other parental breeds. For the simulated data of unrelated breeds, varying f_r_ did not or only very slightly affect the %correct, %incorrect and %unknown, as expected. The main effect of f_r_ was observed for scenarios with closely-related breeds, for which sharing of haplotypes between breeds is more common. Increasing f_r_ mostly allowed to correctly assign a higher percentage of alleles that were previously considered as having an unknown origin. However, the impact of increasing f_r_ from 0 to 10 % on %correct was greater than increasing it from 10 to 20 %. This was also observed for the real data, for which the increase of %correct was less than 1 % when f_r_ increased from 10 to 20 % compared to more than 2 % when f_r_ increased from 0 to 10 %. Based on these results, f_r_ values greater than 0 % were useful and allowed assignment (correctly or incorrectly) of on average more than 90 % of the alleles of a crossbred animal, without (or only slightly) increasing the rate of incorrect assignments (as observed based on simulated data).

### The phasing method

Several phasing methods exist, such as pedigree-based phasing methods (e.g., [25]), LD-based phasing methods (e.g., Beagle [26], SHAPE-IT [27]), and LRP methods [20, 21]. Pedigree-based methods were not considered in this study because the pedigree of crossbred animals is not available in many crossbreeding programs, or their direct parents may not be genotyped. Thus, in real data, purebred and crossbred genotyped animals may be distant relatives that are separated by several generations, the parents of crossbred animals may not be included in the genotype dataset, or the pedigree of crossbred animals could be incomplete. LD-based phasing methods were considered to be suboptimal for crossbred populations because they rely on short haplotypes that may be common to several breeds, as detailed by Hidalgo et al. [28], and Amaral et al. [29], for pig breeds, and by Villa-Angulo et al. [30] for cattle breeds. Both these issues are avoided with LRP methods that aim at identifying and using distant relatives. LRP methods overcome the issue of common LD between breeds because long-range haplotypes are longer than one LD block but are still shared between purebreds and their close crossbred relatives. Also, LRP does not require knowledge of pedigree [21]. AlphaPhase1.1 (version 1) software [20] that implements LRPHLI without pedigree was therefore chosen for this study.

Based on their experience, Hickey et al. [20] recommended the use of core and tail lengths of 300 to 500 SNPs (with a core length of 100 SNPs) for a 60k SNP panel. Consistent with Hickey et al. [20], the longest core and tail lengths required the shortest computational times. Furthermore, computational times increased with increasing distance between breeds for the same core and tail lengths. Distances between breeds were created with the simulation of 5, 20, or 50 generations of random mating before starting the three-way crossbreeding scheme, leading to higher inbreeding levels with increasing distances between breeds. As detailed by Hickey et al. [20], increasing inbreeding levels increases the number of surrogate parents in a dataset, which increases the computational requirements. This is in agreement with the estimated global *F*_ST_ (Table 1). Based on simulated data, the longest core and tail lengths appeared to be more suitable when the breeds were more closely related. Thus, a general recommendation is to increase core and tail lengths as *F*_ST_ decreases, in addition to taking the characteristics of the genome under study into account, such as chromosome lengths and the number of SNP per chromosome.

Increasing the size of the datasets (results not shown) increases computational time. Hickey et al. [20] suggested that the computationally intensive phasing analyses could be performed on a random or selected subset of a large dataset of purebred and crossbred animals. A haplotype library can be built on a subset of data and then used to phase the crossbred animals that were not included in the phased subset. This haplotype library could also be used to phase crossbred individuals that are added to the dataset later on.

The analyses in this study were performed without pedigree for both purebred and crossbred animals. However, in real field data, pedigree may be known for, at least, some animals, e.g., for the purebred animals and this could be considered for the phasing analyses to reduce computation time for the larger datasets [20]. Although not tested in our study, inclusion of pedigree is not expected to improve the percentages of phased and assigned alleles because Hickey et al. [20] reported negligible effects on the phasing performance when pedigree information was ignored.

### Applications

In the context of genomic selection for crossbred performance, the BOA approach can be used to determine the breed origin of alleles at genotyped SNPs for crossbred animals, which is required for models that take breed-specific effects into account (e.g., [3, 5, 31]). The BOA approach could also be useful to perform GWAS based on crossbred performance and taking into consideration that the effects of causative mutations on phenotypes may depend on breed origin. However, future studies are required to evaluate the effects of the low percentages of unknown and incorrect allele assignments on accuracy and bias of genomic predictions (or GWAS).

It should also be noted that some animals had a high percentage of unassigned alleles, which makes them not useful for subsequent analyses. These genotypes could be discarded, or breed origin could be assigned to their alleles based on, e.g. allele frequencies, as proposed above. This latter option should be applied with care, and after exploring the possible reasons for the high percentage of unassigned alleles (e.g., low percentage of phased alleles, breed composition errors, genotype errors).

Some studies have suggested that the use of haplotypes could lead to higher prediction accuracies than using SNP genotypes (e.g., [32, 33]). Potential reasons are that haplotypes may be in higher LD with causative mutations than individual SNPs and, therefore, capture more variation than SNPs. Thus, assigned haplotypes could be used in a haplotype-based genomic model that takes their breed origin into account. Using assigned haplotypes instead of assigned alleles could potentially reduce effects of incorrect allelic assignments.

## Conclusions

The BOA approach accurately assigns breed origin to alleles of crossbred animals in a two- or three-way crossbreeding program. This procedure requires no prior knowledge of pedigree and no close relationships between crossbred and purebred animals, since it relies on long-range phasing.

## Appendix

### Pseudo-code assigning a breed origin to each allele of each SNP

It is assumed that all genotypes of both purebred and crossbred animals were previously phased, and that a library of haplotypes that are assigned to breed origin is available. This library is obtained from phased information on purebred animals (see the section ‘Assigning a breed origin to haplotypes’ for more details). The following notation was used for the pseudo-code:

### Additional rules to the BOA approach

The additional rules to the BOA approach can be applied to assign breed origin to an unassigned allele of a SNP for which the second allele was previously assigned a breed origin, and to assign breed origin to unassigned alleles that were surrounded by alleles that were assigned to the same breed origin. The pseudo-code for the additional rules can be written as follows:

## Additional files

10.1186/s12711-016-0240-y **Table S1**: Percentages of alleles correctly assigned a breed origin (%correct), incorrectly assigned (%incorrect), or unassigned (%unknown) for a BC crossbred animal for the chromosome 1. Results are averages (SD) across the 10 replicates. **Table S2**: Percentages of alleles correctly assigned a breed origin (%correct), incorrectly assigned (%incorrect), or unassigned (%unknown) for a BC crossbred animal for the chromosome 2. Results are averages (SD) across the 10 replicates. **Table S3**: Percentages of alleles correctly assigned a breed origin (%correct), incorrectly assigned (%incorrect), or unassigned (%unknown) for a A(BC) crossbred animal for chromosome 1. Results are averages (SD) across the 10 replicates. **Table S4**: Percentages of alleles correctly assigned a breed origin (%correct), incorrectly assigned (%incorrect), or unassigned (%unknown) for a A(BC) crossbred animal for chromosome 2. Results are averages (SD) across the 10 replicates. **Table S5**: Percentages of BC and A(BC) animals having at least 80 % of assigned alleles, and Spearman rank correlations between the order of the phasing analyses obtained from the forward selection and a predefined order of the same phasing analyses for the A(BC) animals, with a relaxation factor equal to 10 %. Results are averages (SD) across the 10 replicates. **Table S6**: Percentages of assigned alleles of the chromosomes SSC2 and SSC18 for an EF or a D(EF) animal, and percentages of EF and D(EF) animals having at least 80 % of assigned alleles, with a relaxation factor equal to 10 %. **Table S7**: Average (SD) percentages of assigned alleles of the chromosomes SSC2 and SSC18 for an EF or a D(EF) animal. **Table S8**: Percentages of alleles correctly assigned a breed origin (%correct) and incorrectly assigned (%incorrect), for the chromosome 2 with 9 phasing analyses with, or without, the additional rules. Results are averages (SD) across the 10 replicates.
10.1186/s12711-016-0240-y **Figure S1**: Percentages of correct allelic assignments with a relaxation factor equal to 20 % when breeds are distantly related. Minimum (grey) and maximum (black) percentages of correct allelic assignments, for the chromosome 2 and averaged across A(BC) animals of distantly related breeds, as a function of the number of offset and non-offset phasing analyses selected based on a forward selection. Reported results are averages and SD across all replicates. **Figure S2**: Percentages of correct allelic assignments with a relaxation factor equal to 0 % when breeds are distantly related. Minimum (grey) and maximum (black) percentages of correct allelic assignments, for the chromosome 2 and averaged across A(BC) animals of distantly related breeds, as a function of the number of offset and non-offset phasing analyses selected based on a forward selection. Reported results are averages and SD across all replicates. **Figure S3**: Percentages of correct allelic assignments with a relaxation factor equal to 20 % when breeds are unrelated. Minimum (grey) and maximum (black) percentages of correct allelic assignments, for the chromosome 2 and averaged across A(BC) animals of unrelated breeds, as a function of the number of offset and non-offset phasing analyses selected based on a forward selection. Reported results are averages and SD across all replicates. **Figure S4**: Breed origin of the two alleles for each SNP of the chromosome SSC2 in 20 EF animals using BOA and the additional rules. Alleles from breeds E and F are in green and blue, respectively. Grey regions are unassigned alleles. Relaxation factor was equal to 0 %. **Figure S5**: Breed origin of the two alleles for each SNP of the chromosome SSC2 in 20 D(EF) animals using BOA and the additional rules. Alleles from breeds D, E, and F are in brown, green, and blue, respectively. Grey regions are unassigned alleles. Relaxation factor was equal to 0 %.
