# Supplementary material for: Assigning breed origin to alleles in crossbred animals
Source: Genet Sel Evol. 2016 Aug 22;48(1):61. doi: 10.1186/s12711-016-0240-y (PMC4994281; doi:10.1186/s12711-016-0240-y)
Supplement: Supplementary file 1 — 10.1186/s12711-016-0240-y Table S1: Percentages of alleles correctly assigned a breed origin (%correct), incorrectly assigned (%incorrect), or unassigned (%unknown) for a BC crossbred animal for the chromosome 1. Results are averages (SD) across the 10 replicates. Table S2: Percentages of alleles correctly assigned a breed origin (%correct), incorrectly assigned (%incorrect), or unassigned (%unknown) for a BC crossbred animal for the chromosome 2. Results are averages (SD) across the 10 replicates. Table S3: Percentages of alleles correctly assigned a breed origin (%correct), incorrectly assigned (%incorrect), or unassigned (%unknown) for a A(BC) crossbred animal for chromosome 1. Results are averages (SD) across the 10 replicates. Table S4: Percentages of alleles correctly assigned a breed origin (%correct), incorrectly assigned (%incorrect), or unassigned (%unknown) for a A(BC) crossbred animal for chromosome 2. Results are averages (SD) across the 10 replicates. Table S5: Percentages of BC and A(BC) animals having at least 80 % of assigned alleles, and Spearman rank correlations between the order of the phasing analyses obtained from the forward selection and a predefined order of the same phasing analyses for the A(BC) animals, with a relaxation factor equal to 10 %. Results are averages (SD) across the 10 replicates. Table S6: Percentages of assigned alleles of the chromosomes SSC2 and SSC18 for an EF or a D(EF) animal, and percentages of EF and D(EF) animals having at least 80 % of assigned alleles, with a relaxation factor equal to 10 %. Table S7: Average (SD) percentages of assigned alleles of the chromosomes SSC2 and SSC18 for an EF or a D(EF) animal. Table S8: Percentages of alleles correctly assigned a breed origin (%correct) and incorrectly assigned (%incorrect), for the chromosome 2 with 9 phasing analyses with, or without, the additional rules. Results are averages (SD) across the 10 replicates. [file 12711_2016_240_MOESM1_ESM.pdf]

Table S1. Percentages of alleles correctly assigned a breed origin (%correct), incorrectly assigned (%incorrect), or unassigned (%unknown) for a BC crossbred animal for the chromosome 1. Results are averages (SD) across the 10 replicates.

Closely related breeds									
f_r[1]	%correct			%incorrect			%unknown		
	Average	Min	Max	Average	Min	Max	Average	Min	Max
20	95.15 (0.37)	77.00 (5.19)	99.99 (0.03)	0.39 (0.02)	0.00 (0.00)	2.99 (0.86)	4.46 (0.37)	0.01 (0.02)	22.38 (5.20)
10	94.60 (0.40)	75.76 (4.38)	99.98 (0.03)	0.37 (0.03)	0.00 (0.00)	2.66 (0.87)	5.02 (0.40)	0.01 (0.02)	23.50 (4.52)
0	92.42 (0.57)	72.55 (4.31)	99.92 (0.12)	0.36 (0.02)	0.00 (0.00)	2.49 (0.36)	7.22 (0.56)	0.06 (0.12)	26.69 (4.16)
Distantly related breeds									
f_r	%correct			%incorrect			%unknown		
	Average	Min	Max	Average	Min	Max	Average	Min	Max
20	98.09 (0.09)	89.50 (0.80)	100.00 (0.00)	0.32 (0.02)	0.00 (0.00)	2.12 (0.43)	1.60 (0.08)	0.00 (0.00)	9.56 (1.12)
10	98.08 (0.12)	89.65 (0.95)	100.00 (0.00)	0.31 (0.02)	0.00 (0.00)	2.12 (0.43)	1.60 (0.10)	0.00 (0.00)	9.43 (1.13)
0	97.92 (0.22)	88.91 (1.12)	100.00 (0.00)	0.30 (0.02)	0.00 (0.00)	2.05 (0.46)	1.77 (0.21)	0.00 (0.00)	10.30 (1.27)
Unrelated breeds									
f_r	%correct			%incorrect			%unknown		
	Average	Min	Max	Average	Min	Max	Average	Min	Max
20	98.58 (0.13)	91.58 (1.58)	100.00 (0.00)	0.28 (0.04)	0.00 (0.00)	1.59 (0.32)	1.14 (0.11)	0.00 (0.00)	7.56 (1.36)
10	98.58 (0.13)	91.58 (1.58)	100.00 (0.00)	0.28 (0.04)	0.00 (0.00)	1.59 (0.32)	1.14 (0.11)	0.00 (0.00)	7.56 (1.36)
0	98.58 (0.14)	91.55 (1.56)	100.00 (0.00)	0.28 (0.04)	0.00 (0.00)	1.59 (0.32)	1.14 (0.11)	0.00 (0.00)	7.56 (1.36)

[1]f_r = relaxation factor

Table S2. Percentages of alleles correctly assigned a breed origin (%correct), incorrectly assigned (%incorrect), or unassigned (%unknown) for a BC crossbred animal for the chromosome 2. Results are averages (SD) across the 10 replicates.

Closely related breeds									
f_r[1]	%correct			%incorrect			%unknown		
	Average	Min	Max	Average	Min	Max	Average	Min	Max
20	94.05 (0.71)	45.18 (9.56)	100.00 (0.00)	0.47 (0.07)	0.00 (0.00)	8.90 (3.33)	5.48 (0.68)	0.00 (0.00)	53.47 (8.84)
10	92.95 (0.91)	40.12 (11.64)	100.00 (0.00)	0.46 (0.06)	0.00 (0.00)	10.09 (3.58)	6.59 (0.88)	0.00 (0.00)	58.64 (10.79)
0	90.72 (1.32)	32.44 (15.26)	100.00 (0.00)	0.45 (0.05)	0.00 (0.00)	8.91 (2.85)	8.83 (1.31)	0.00 (0.00)	66.96 (15.52)
Distantly related breeds									
f_r	%correct			%incorrect			%unknown		
	Average	Min	Max	Average	Min	Max	Average	Min	Max
20	97.62 (0.39)	71.72 (7.01)	100.00 (0.00)	0.38 (0.06)	0.00 (0.00)	6.60 (1.68)	2.00 (0.35)	0.00 (0.00)	26.16 (5.80)
10	97.56 (0.36)	71.15 (6.71)	100.00 (0.00)	0.38 (0.06)	0.00 (0.00)	6.55 (1.70)	2.06 (0.32)	0.00 (0.00)	26.82 (5.56)
0	97.44 (0.39)	69.11 (6.85)	100.00 (0.00)	0.37 (0.06)	0.00 (0.00)	6.33 (1.83)	2.19 (0.35)	0.00 (0.00)	29.22 (5.91)
Unrelated breeds									
f_r	%correct			%incorrect			%unknown		
	Average	Min	Max	Average	Min	Max	Average	Min	Max
20	98.00 (0.48)	72.27 (11.22)	100.00 (0.00)	0.36 (0.11)	0.00 (0.00)	4.25 (1.07)	1.64 (0.42)	0.00 (0.00)	26.03 (10.98)
10	98.00 (0.49)	72.27 (11.22)	100.00 (0.00)	0.36 (0.11)	0.00 (0.00)	4.25 (1.07)	1.64 (0.42)	0.00 (0.00)	26.03 (10.98)
0	98.00 (0.49)	72.27 (11.22)	100.00 (0.00)	0.35 (0.11)	0.00 (0.00)	3.95 (0.74)	1.65 (0.43)	0.00 (0.00)	26.03 (10.98)

[1]f_r = relaxation factor

Table S3. Percentages of alleles correctly assigned a breed origin (%correct), incorrectly assigned (%incorrect), or unassigned (%unknown) for a A(BC) crossbred animal for the chromosome 1. Results are averages (SD) across the 10 replicates.

Closely related breeds									
f_r[1]	%correct			%incorrect			%unknown		
	Average	Min	Max	Average	Min	Max	Average	Min	Max
20	93.18 (0.43)	76.17 (3.40)	99.77 (0.21)	1.69 (0.10)	0.00 (0.00)	8.96 (1.56)	5.13 (0.41)	0.07 (0.07)	19.71 (3.23)
10	92.24 (0.52)	73.15 (3.93)	99.72 (0.28)	1.57 (0.10)	0.00 (0.00)	8.25 (1.69)	6.19 (0.49)	0.12 (0.13)	22.76 (4.18)
0	88.91 (0.63)	66.00 (4.21)	99.18 (0.58)	1.58 (0.10)	0.00 (0.00)	8.47 (1.33)	9.51 (0.59)	0.51 (0.38)	31.48 (3.78)
Distantly related breeds									
f_r	%correct			%incorrect			%unknown		
	Average	Min	Max	Average	Min	Max	Average	Min	Max
20	96.21 (0.16)	85.97 (1.60)	99.93 (0.06)	0.96 (0.09)	0.00 (0.00)	4.98 (0.84)	2.83 (0.11)	0.03 (0.04)	11.46 (1.07)
10	96.18 (0.17)	86.29 (1.29)	99.93 (0.06)	0.95 (0.09)	0.00 (0.00)	4.79 (0.57)	2.88 (0.12)	0.03 (0.04)	11.48 (1.08)
0	95.92 (0.23)	85.91 (1.52)	99.92 (0.05)	0.95 (0.10)	0.00 (0.00)	4.78 (0.67)	3.13 (0.18)	0.03 (0.04)	12.09 (0.96)
Unrelated breeds									
f_r	%correct			%incorrect			%unknown		
	Average	Min	Max	Average	Min	Max	Average	Min	Max
20	96.99 (0.15)	88.36 (1.32)	99.97 (0.03)	0.68 (0.04)	0.00 (0.00)	3.19 (0.72)	2.33 (0.14)	0.01 (0.02)	9.99 (1.83)
10	96.99 (0.16)	88.36 (1.32)	99.97 (0.03)	0.68 (0.04)	0.00 (0.00)	3.19 (0.72)	2.33 (0.14)	0.01 (0.02)	9.99 (1.83)
0	96.97 (0.15)	88.35 (1.31)	99.97 (0.03)	0.69 (0.04)	0.00 (0.00)	3.21 (0.70)	2.34 (0.14)	0.01 (0.02)	10.14 (1.69)

[1] f_r = relaxation factor

Table S4. Percentages of alleles correctly assigned a breed origin (%correct), incorrectly assigned (%incorrect), or unassigned (%unknown) for a A(BC) crossbred animal for the chromosome 2. Results are averages (SD) across the 10 replicates.

Closely related breeds									
f_r[1]	%correct			%incorrect			%unknown		
	Average	Min	Max	Average	Min	Max	Average	Min	Max
20	91.64 (0.95)	41.19 (8.70)	100.00 (0.00)	1.99 (0.17)	0.00 (0.00)	27.62 (6.67)	6.37 (0.91)	0.00 (0.00)	54.24 (10.13)
10	90.44 (1.00)	36.44 (8.70)	100.00 (0.00)	1.86 (0.14)	0.00 (0.00)	24.66 (4.33)	7.69 (0.98)	0.00 (0.00)	60.04 (11.53)
0	87.37 (1.55)	28.74 (8.73)	100.00 (0.00)	1.83 (0.17)	0.00 (0.00)	24.18 (4.61)	10.80 (1.47)	0.00 (0.00)	66.40 (9.10)
Distantly related breeds									
f_r	%correct			%incorrect			%unknown		
	Average	Min	Max	Average	Min	Max	Average	Min	Max
20	95.51 (0.50)	64.63 (7.80)	100.00 (0.00)	1.14 (0.15)	0.00 (0.00)	15.66 (4.72)	3.35 (0.42)	0.00 (0.00)	32.28 (8.57)
10	95.45 (0.49)	64.63 (7.80)	100.00 (0.00)	1.12 (0.15)	0.00 (0.00)	15.53 (4.77)	3.44 (0.40)	0.00 (0.00)	32.28 (8.57)
0	95.11 (0.48)	63.46 (8.07)	100.00 (0.00)	1.09 (0.15)	0.00 (0.00)	16.16 (4.55)	3.79 (0.39)	0.00 (0.00)	33.51 (8.69)
Unrelated breeds									
f_r	%correct			%incorrect			%unknown		
	Average	Min	Max	Average	Min	Max	Average	Min	Max
20	95.83 (0.65)	64.90 (12.76)	100.00 (0.00)	0.87 (0.14)	0.00 (0.00)	11.28 (1.77)	3.30 (0.59)	0.00 (0.00)	32.24 (11.40)
10	95.81 (0.66)	64.90 (12.76)	100.00 (0.00)	0.87 (0.14)	0.00 (0.00)	11.28 (1.77)	3.32 (0.60)	0.00 (0.00)	32.24 (11.40)
0	95.78 (0.66)	64.20 (12.85)	100.00 (0.00)	0.87 (0.14)	0.00 (0.00)	11.58 (1.62)	3.35 (0.60)	0.00 (0.00)	32.83 (11.53)

[1] f_r = relaxation factor

Table S5. Percentages of BC and A(BC) animals having at least 80% of assigned alleles, and Spearman rank correlations between the order of the phasing analyses obtained from the forward selection and a predefined order of the same phasing analyses for the A(BC) animals, with a relaxation factor equal to 10%. Results are averages (SD) across the 10 replicates.

Scenarios	BC animals		A(BC) animals		Rank correlations	
	Chr. 1	Chr. 2	Chr. 1	Chr. 2	Chr. 1	Chr. 2
Closely related breeds	99.52	90.56	99.53	89.23	0.37	0.23
	(0.51)	(2.28)	(0.58)	(2.39)	(0.17)	(0.16)
Distantly related breeds	100.00	99.23	100.00	98.48	0.04	-0.20
	(0.00)	(0.75)	(0.00)	(1.06)	(0.16)	(0.15)
Unrelated breeds	100.00	99.57	100.00	98.04	-0.23	-0.13
	(0.00)	(0.56)	(0.00)	(1.16)	(0.08)	(0.23)

Table S6. Percentages of assigned alleles of the chromosomes SSC2 and SSC18 for an EF or a D(EF) animal, and percentages of EF and D(EF) animals having at least 80% of assigned alleles, with a relaxation factor equal to 10%.

Chromosome	%assigned				Percentage of animals with >=80%assigned
	Average	SD	Min	Max	
SSC2					
EF	92.18	8.31	34.86	100.00	93.82
D(EF)	92.39	8.64	44.60	99.98	96.68
SSC18					
EF	90.18	11.10	24.23	100.00	84.57
D(EF)	90.02	11.18	45.21	100.00	82.57

Table S7. Average (SD) percentages of assigned alleles of the chromosomes SSC2 and SSC18 for an EF or a D(EF) animal.

Chromosome	Relaxation factor	Breed D	Breed E	Breed F
SSC2				
EF	10	-	48.17 (4.34)	44.09 (6.50)
	0	-	46.48 (5.38)	42.42 (6.88)
D(EF)	10	47.10 (7.16)	23.87 (17.66)	21.42 (16.97)
	0	45.83 (7.65)	23.02 (17.14)	20.73 (16.36)
SSC18				
EF	10	-	47.09 (6.97)	43.08 (8.58)
	0	-	46.53 (7.34)	42.23 (8.94)
D(EF)	10	47.51 (7.68)	21.70 (19.16)	20.82 (17.96)
	0	47.32 (7.79)	20.92 (18.85)	20.31 (17.68)

Table S8. Percentages of alleles correctly assigned a breed origin (%correct) and incorrectly assigned (%incorrect), for the chromosome 2 with 9 phasing analyses with, or without, the additional rules. Results are averages (SD) across the 10 replicates.

Relaxation factor	Additional rules	BC animals		A(BC) animals	
		Average %correct	Average %incorrect	Average %correct	Average %incorrect
20	No	94.05 (0.71)	0.47 (0.07)	91.64 (0.95)	1.99 (0.17)
	Yes	98.80 (0.20)	0.85 (0.10)	93.44 (0.63)	2.24 (0.19)
0	No	90.72 (1.32)	0.45 (0.05)	87.37 (1.55)	1.83 (0.17)
	Yes	98.32 (1.05)	0.84 (0.08)	91.06 (1.05)	2.05 (0.19)
