# Supplementary figures and images for: Assigning breed origin to alleles in crossbred animals
Source: Genet Sel Evol. 2016 Aug 22;48(1):61. doi: 10.1186/s12711-016-0240-y (PMC4994281; doi:10.1186/s12711-016-0240-y)

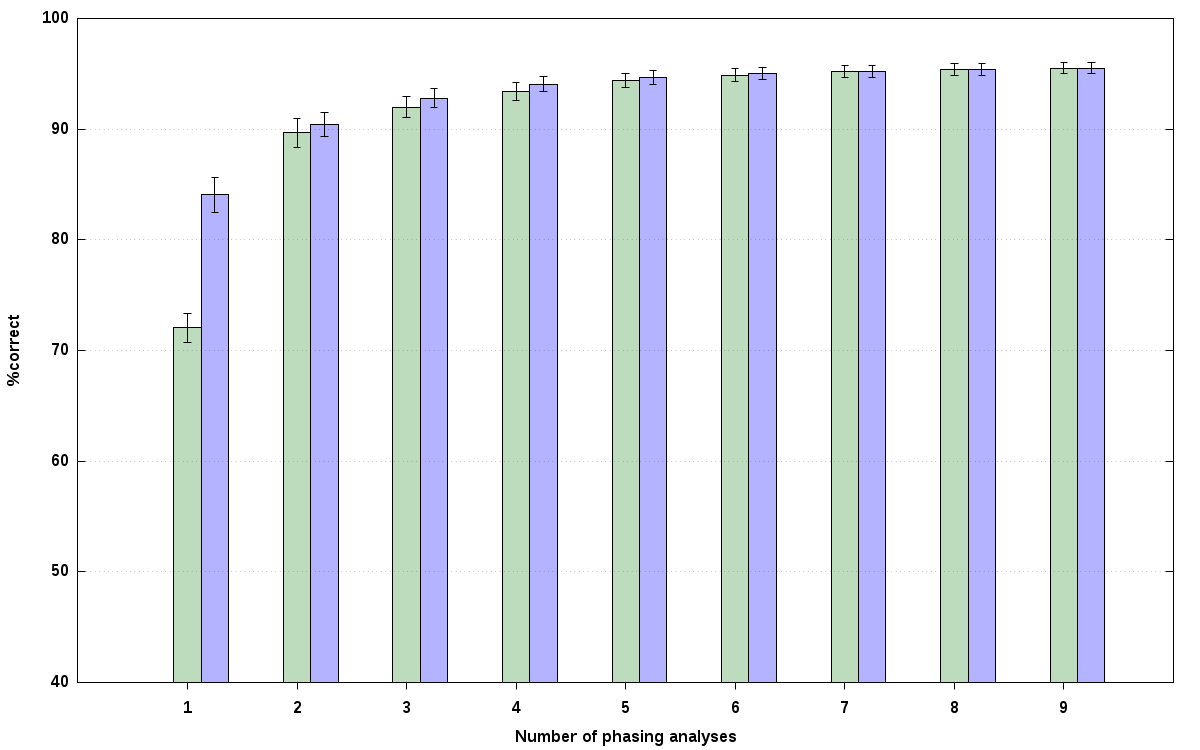

Supplement: Supplementary file 2 — 10.1186/s12711-016-0240-y Figure S1: Percentages of correct allelic assignments with a relaxation factor equal to 20 % when breeds are distantly related. Minimum (grey) and maximum (black) percentages of correct allelic assignments, for the chromosome 2 and averaged across A(BC) animals of distantly related breeds, as a function of the number of offset and non-offset phasing analyses selected based on a forward selection. Reported results are averages and SD across all replicates. Figure S2: Percentages of correct allelic assignments with a relaxation factor equal to 0 % when breeds are distantly related. Minimum (grey) and maximum (black) percentages of correct allelic assignments, for the chromosome 2 and averaged across A(BC) animals of distantly related breeds, as a function of the number of offset and non-offset phasing analyses selected based on a forward selection. Reported results are averages and SD across all replicates. Figure S3: Percentages of correct allelic assignments with a relaxation factor equal to 20 % when breeds are unrelated. Minimum (grey) and maximum (black) percentages of correct allelic assignments, for the chromosome 2 and averaged across A(BC) animals of unrelated breeds, as a function of the number of offset and non-offset phasing analyses selected based on a forward selection. Reported results are averages and SD across all replicates. Figure S4: Breed origin of the two alleles for each SNP of the chromosome SSC2 in 20 EF animals using BOA and the additional rules. Alleles from breeds E and F are in green and blue, respectively. Grey regions are unassigned alleles. Relaxation factor was equal to 0 %. Figure S5: Breed origin of the two alleles for each SNP of the chromosome SSC2 in 20 D(EF) animals using BOA and the additional rules. Alleles from breeds D, E, and F are in brown, green, and blue, respectively. Grey regions are unassigned alleles. Relaxation factor was equal to 0 %. [file 12711_2016_240_MOESM2_ESM.zip › additional_file_2/FigureS1.png]

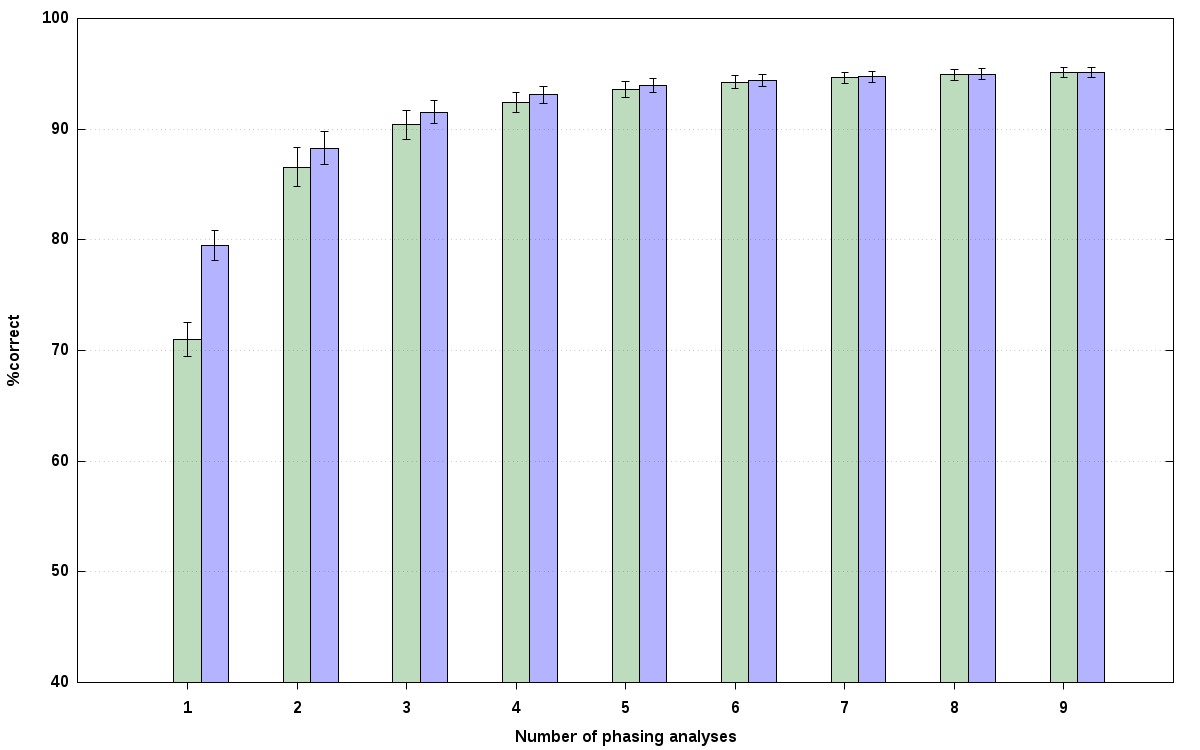

Supplement: Supplementary file 2 — 10.1186/s12711-016-0240-y Figure S1: Percentages of correct allelic assignments with a relaxation factor equal to 20 % when breeds are distantly related. Minimum (grey) and maximum (black) percentages of correct allelic assignments, for the chromosome 2 and averaged across A(BC) animals of distantly related breeds, as a function of the number of offset and non-offset phasing analyses selected based on a forward selection. Reported results are averages and SD across all replicates. Figure S2: Percentages of correct allelic assignments with a relaxation factor equal to 0 % when breeds are distantly related. Minimum (grey) and maximum (black) percentages of correct allelic assignments, for the chromosome 2 and averaged across A(BC) animals of distantly related breeds, as a function of the number of offset and non-offset phasing analyses selected based on a forward selection. Reported results are averages and SD across all replicates. Figure S3: Percentages of correct allelic assignments with a relaxation factor equal to 20 % when breeds are unrelated. Minimum (grey) and maximum (black) percentages of correct allelic assignments, for the chromosome 2 and averaged across A(BC) animals of unrelated breeds, as a function of the number of offset and non-offset phasing analyses selected based on a forward selection. Reported results are averages and SD across all replicates. Figure S4: Breed origin of the two alleles for each SNP of the chromosome SSC2 in 20 EF animals using BOA and the additional rules. Alleles from breeds E and F are in green and blue, respectively. Grey regions are unassigned alleles. Relaxation factor was equal to 0 %. Figure S5: Breed origin of the two alleles for each SNP of the chromosome SSC2 in 20 D(EF) animals using BOA and the additional rules. Alleles from breeds D, E, and F are in brown, green, and blue, respectively. Grey regions are unassigned alleles. Relaxation factor was equal to 0 %. [file 12711_2016_240_MOESM2_ESM.zip › additional_file_2/FigureS2.png]

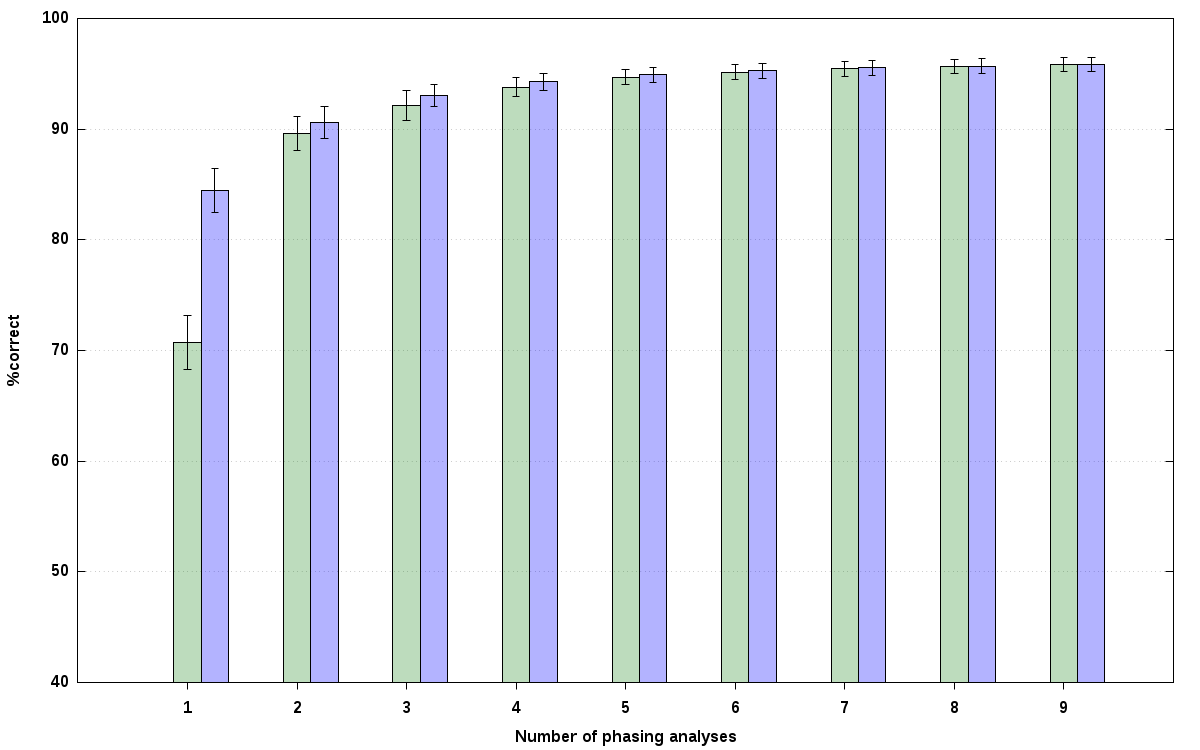

Supplement: Supplementary file 2 — 10.1186/s12711-016-0240-y Figure S1: Percentages of correct allelic assignments with a relaxation factor equal to 20 % when breeds are distantly related. Minimum (grey) and maximum (black) percentages of correct allelic assignments, for the chromosome 2 and averaged across A(BC) animals of distantly related breeds, as a function of the number of offset and non-offset phasing analyses selected based on a forward selection. Reported results are averages and SD across all replicates. Figure S2: Percentages of correct allelic assignments with a relaxation factor equal to 0 % when breeds are distantly related. Minimum (grey) and maximum (black) percentages of correct allelic assignments, for the chromosome 2 and averaged across A(BC) animals of distantly related breeds, as a function of the number of offset and non-offset phasing analyses selected based on a forward selection. Reported results are averages and SD across all replicates. Figure S3: Percentages of correct allelic assignments with a relaxation factor equal to 20 % when breeds are unrelated. Minimum (grey) and maximum (black) percentages of correct allelic assignments, for the chromosome 2 and averaged across A(BC) animals of unrelated breeds, as a function of the number of offset and non-offset phasing analyses selected based on a forward selection. Reported results are averages and SD across all replicates. Figure S4: Breed origin of the two alleles for each SNP of the chromosome SSC2 in 20 EF animals using BOA and the additional rules. Alleles from breeds E and F are in green and blue, respectively. Grey regions are unassigned alleles. Relaxation factor was equal to 0 %. Figure S5: Breed origin of the two alleles for each SNP of the chromosome SSC2 in 20 D(EF) animals using BOA and the additional rules. Alleles from breeds D, E, and F are in brown, green, and blue, respectively. Grey regions are unassigned alleles. Relaxation factor was equal to 0 %. [file 12711_2016_240_MOESM2_ESM.zip › additional_file_2/FigureS3.png]

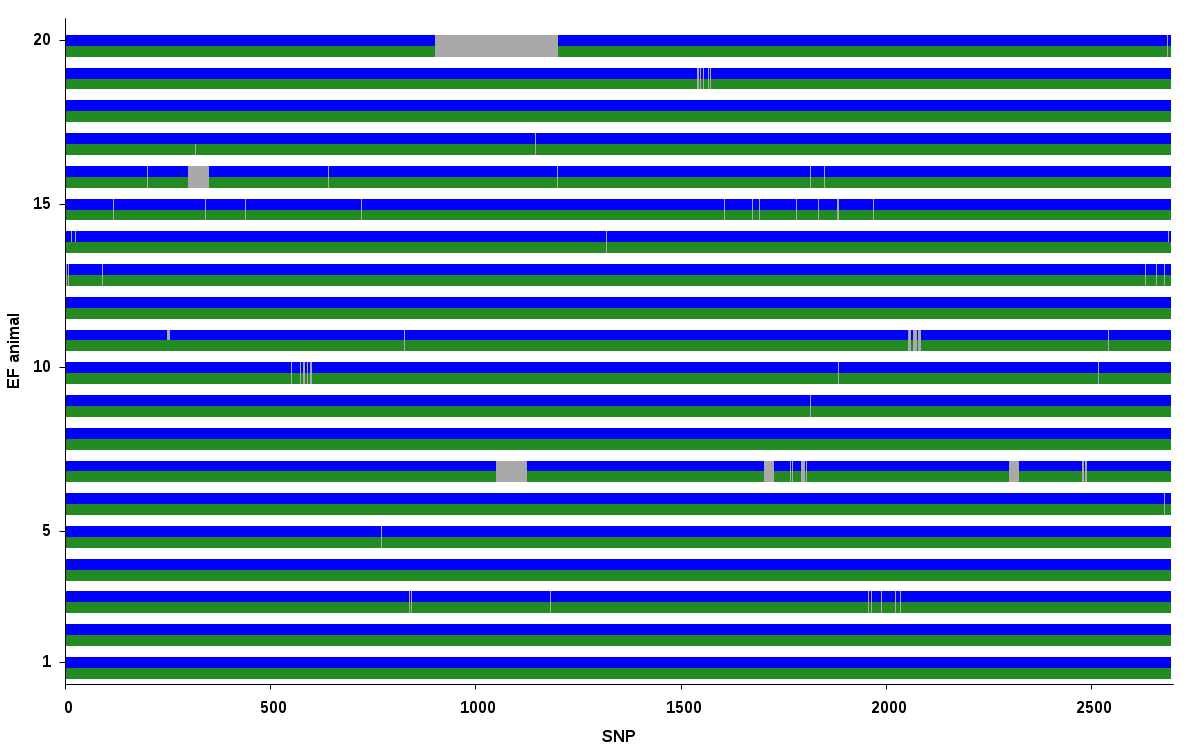

Supplement: Supplementary file 2 — 10.1186/s12711-016-0240-y Figure S1: Percentages of correct allelic assignments with a relaxation factor equal to 20 % when breeds are distantly related. Minimum (grey) and maximum (black) percentages of correct allelic assignments, for the chromosome 2 and averaged across A(BC) animals of distantly related breeds, as a function of the number of offset and non-offset phasing analyses selected based on a forward selection. Reported results are averages and SD across all replicates. Figure S2: Percentages of correct allelic assignments with a relaxation factor equal to 0 % when breeds are distantly related. Minimum (grey) and maximum (black) percentages of correct allelic assignments, for the chromosome 2 and averaged across A(BC) animals of distantly related breeds, as a function of the number of offset and non-offset phasing analyses selected based on a forward selection. Reported results are averages and SD across all replicates. Figure S3: Percentages of correct allelic assignments with a relaxation factor equal to 20 % when breeds are unrelated. Minimum (grey) and maximum (black) percentages of correct allelic assignments, for the chromosome 2 and averaged across A(BC) animals of unrelated breeds, as a function of the number of offset and non-offset phasing analyses selected based on a forward selection. Reported results are averages and SD across all replicates. Figure S4: Breed origin of the two alleles for each SNP of the chromosome SSC2 in 20 EF animals using BOA and the additional rules. Alleles from breeds E and F are in green and blue, respectively. Grey regions are unassigned alleles. Relaxation factor was equal to 0 %. Figure S5: Breed origin of the two alleles for each SNP of the chromosome SSC2 in 20 D(EF) animals using BOA and the additional rules. Alleles from breeds D, E, and F are in brown, green, and blue, respectively. Grey regions are unassigned alleles. Relaxation factor was equal to 0 %. [file 12711_2016_240_MOESM2_ESM.zip › additional_file_2/FigureS4.png]

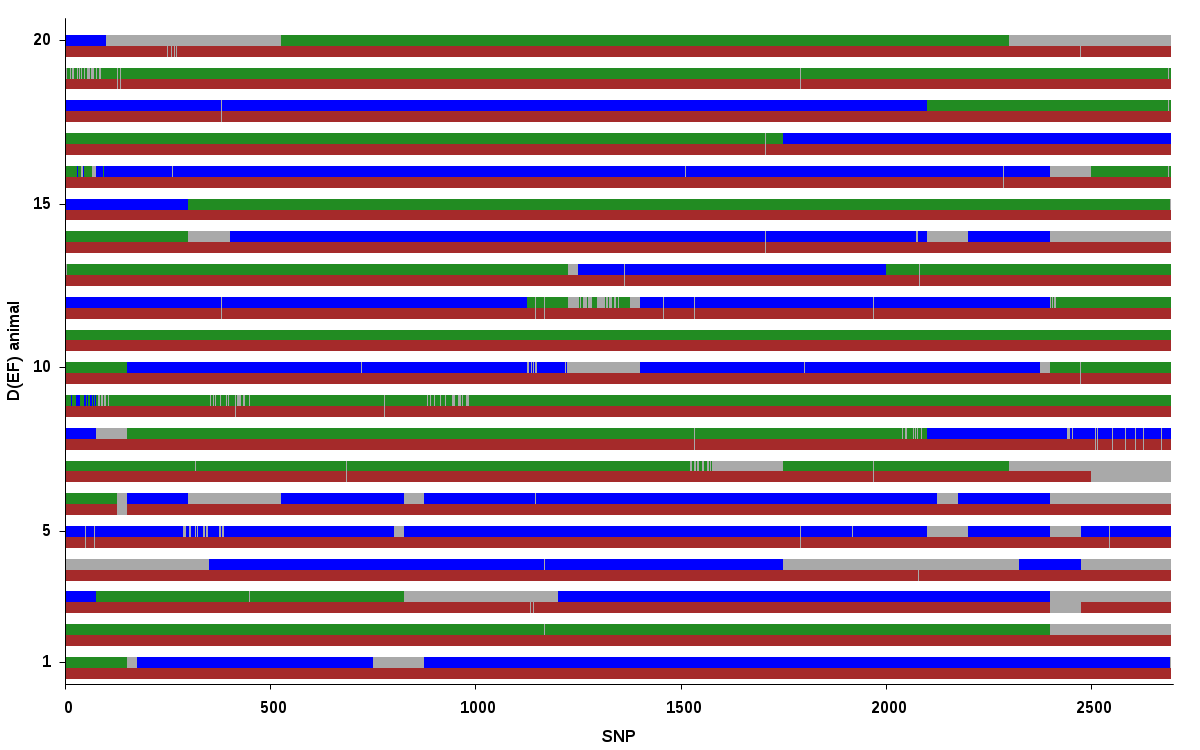

Supplement: Supplementary file 2 — 10.1186/s12711-016-0240-y Figure S1: Percentages of correct allelic assignments with a relaxation factor equal to 20 % when breeds are distantly related. Minimum (grey) and maximum (black) percentages of correct allelic assignments, for the chromosome 2 and averaged across A(BC) animals of distantly related breeds, as a function of the number of offset and non-offset phasing analyses selected based on a forward selection. Reported results are averages and SD across all replicates. Figure S2: Percentages of correct allelic assignments with a relaxation factor equal to 0 % when breeds are distantly related. Minimum (grey) and maximum (black) percentages of correct allelic assignments, for the chromosome 2 and averaged across A(BC) animals of distantly related breeds, as a function of the number of offset and non-offset phasing analyses selected based on a forward selection. Reported results are averages and SD across all replicates. Figure S3: Percentages of correct allelic assignments with a relaxation factor equal to 20 % when breeds are unrelated. Minimum (grey) and maximum (black) percentages of correct allelic assignments, for the chromosome 2 and averaged across A(BC) animals of unrelated breeds, as a function of the number of offset and non-offset phasing analyses selected based on a forward selection. Reported results are averages and SD across all replicates. Figure S4: Breed origin of the two alleles for each SNP of the chromosome SSC2 in 20 EF animals using BOA and the additional rules. Alleles from breeds E and F are in green and blue, respectively. Grey regions are unassigned alleles. Relaxation factor was equal to 0 %. Figure S5: Breed origin of the two alleles for each SNP of the chromosome SSC2 in 20 D(EF) animals using BOA and the additional rules. Alleles from breeds D, E, and F are in brown, green, and blue, respectively. Grey regions are unassigned alleles. Relaxation factor was equal to 0 %. [file 12711_2016_240_MOESM2_ESM.zip › additional_file_2/FigureS5.png]
